# Peripheral lysosome levels dictate mTORC1 inactivation even when catabolically impaired

**DOI:** 10.1186/s12964-026-02659-9

**Published:** 2026-01-20

**Authors:** Huy Quang Dang, Therése Forssén, Spyridon Pantelios, Aisegkioul Nteli Chatzioglou, Ewa Kurzejamska, C. Theresa Vincent, Yasir Ibrahim, Anders P. Mutvei

**Affiliations:** 1 Department of Laboratory Medicine, Division of Pathology, Karolinska Institutet, Stockholm, Sweden; 2 Araucaria Laboratories, Inc., New York, USA; 3 Department of Microbiology, NYU Grossman School of Medicine, New York, NY 10016 USA

**Keywords:** MTORC1, Amino acid deprivation, Lysosome positioning, PI3K-Akt signaling, Catabolically impaired lysosomes, Rap1, Rab7

## Abstract

The mechanistic target of rapamycin complex 1 (mTORC1) is a central driver of cell growth that is frequently hyperactivated in cancer. While mTORC1 is activated at the lysosomal surface in response to growth factors and amino acids, the processes governing its inactivation are not fully understood. Here, we report that sustained mTORC1 suppression during leucine or arginine starvation requires the translocation of peripheral lysosomes to the perinuclear region. Our data suggest that a pool of mTOR remains active at peripheral lysosomes during starvation, and that increased spatial separation between lysosomes and the plasma membrane attenuates PI3K/Akt signaling—thereby reducing inputs that otherwise maintain mTORC1 activity. Consequently, preventing lysosome translocation and increasing peripheral lysosome levels sustains mTORC1 signaling during prolonged starvation in a PI3K/Akt-dependent manner independently of autophagy. Under these conditions, mTORC1 signaling persists even when lysosomal catabolism is perturbed by chloroquine or concanamycin A. Collectively, these data indicate that the peripheral lysosome pool, even when catabolically impaired, can sustain mTORC1 signaling under nutrient scarcity, by modulating PI3K/Akt signaling input to the pathway. These observations identify peripheral lysosome levels as a critical determinant of mTORC1 inactivation during nutrient stress and may have implications for diseases with aberrant mTORC1 signaling, including cancer.

**Supplementary Information:**

The online version contains supplementary material available at 10.1186/s12964-026-02659-9.

## Background

The serine/threonine kinase mechanistic target of rapamycin complex 1 (mTORC1) is a central regulator of cell growth that is hyperactivated in multiple diseases including cancer [1, 2]. In response to growth factors and amino acids, mTORC1 is activated at the surface of late endosomes and lysosomes (subsequently referred to as lysosomes) through distinct upstream signaling mechanisms [3–5]. Growth factor receptor activation by ligand binding initiates PI3K/Akt signaling, leading to the inactivation of tuberous sclerosis complex (TSC) component TSC2 and the activation of the Rheb GTPase, a canonical activator of mTORC1 that is associated with lysosomes [6–9]. In contrast, amino acid signaling is predominantly transmitted independently of TSC2 and Rheb through a process reliant on Rag GTPases, which regulate the recruitment and release of mTORC1 on the lysosomal surface [10–13]. Amino acid signaling additionally requires the vacuolar ATPase (v-ATPase) [14], also essential for acidifying the lysosome lumen [15]. Once activated, mTORC1 promotes an array of anabolic events, including protein translation and lipid synthesis, while suppressing catabolic processes such as macroautophagy (hereafter referred to as autophagy) in which lysosomes recycle macromolecules after fusion with autophagosomes [16].

As our understanding of lysosome-mediated mTORC1 activation has expanded [1, 2], it has become increasingly clear that the intracellular positioning of lysosomes, which ranges from the perinuclear region to the peripheries near the plasma membrane, determines their potential to activate mTORC1 signaling [15, 17–19]. Cellular signaling initiated by growth factors must traverse the cytoplasm to reach lysosomes to activate mTORC1 [20, 21]. As a result, peripheral lysosomes, due to their proximity to the plasma membrane where growth-factor receptors reside, exhibit a markedly enhanced ability to induce mTORC1 signaling compared to their perinuclear counterparts upon stimulation with growth factors and nutrients [22–24]. Moreover, redirecting lysosomes toward the plasma membrane resulted in mTORC1 hyperactivation, consistent with intensified growth factor input under these conditions [23].

In contrast, whether lysosomal positioning determines mTORC1 inactivation upon nutrient starvation remains unclear. It is well documented that total amino acid depletion induces translocation of peripheral lysosomes to the perinuclear region, rapidly reducing their peripheral lysosome localization [23, 25–27]. However, given that total amino acid starvation inactivates mTORC1 independently of lysosome spatial organization, by dissociating mTOR from lysosomes [13], the extent to which lysosome distancing from stimulatory growth factor signals contributes to mTORC1 suppression remains poorly understood. We therefore analyze pathway inactivation during leucine or arginine starvation alone, since these amino acids are pivotal for mTORC1 signaling [28], and leucine starvation has been reported not to fully dissociate mTOR from lysosomes [29].

Here, we demonstrate that sustained mTORC1 repression during leucine or arginine starvation requires translocation of peripheral lysosomes to the perinuclear region. While starvation acutely suppresses mTORC1 through Rag GTPase-dependent signaling, this effect is partial, with a pool of mTORC1 remaining activated at lysosomes under prolonged starvation that is susceptible to changes in lysosome positioning. We conclude that sustained mTORC1 inactivation requires lysosome distancing from the plasma membrane to reduce input from stimulatory PI3K/Akt signaling. Consistently, impairing lysosome translocation and elevating peripheral lysosome levels drive mTORC1 signaling during long-term starvation in a PI3K/Akt-dependent, autophagy-independent manner. Under these conditions, mTORC1 is also strikingly resistant to lysosomal catabolic inhibition by chloroquine (CQ) or concanamycin A (ConA), indicating that peripheral lysosomes can sustain mTORC1 signaling even when catabolically impaired. Collectively, our results indicate that increased peripheral lysosome abundance can sustain mTORC1 signaling independently of nutrient regeneration from autophagy. These findings may be relevant in diseases with dysregulated cell growth, such as cancer, where peripheral lysosome positioning and mTORC1 signaling are elevated [30–32].

## Materials and methods

### Cell culture

HEK293A, U2OS, HEK293A-dnATG4 and HEK293A RagA/B knockout cells were cultured in high-glucose DMEM (41966; Gibco) with 10% Fetal Bovine Serum (FBS; Gibco) and 1% Penicillin–Streptomycin (Gibco). For starvation experiments, cells were cultured in high-glucose DMEM with 10% dialyzed FBS (Gibco) containing either all amino acids (control) or all amino acids except leucine or arginine.

RagA/B CRISPR/Cas9 knockout cells were a kind gift from Drs. Kun-Liang Guan and Jenna L. Jewell [33]. HEK293A cells stably expressing dnATG4 were generated by transfection with pmStrawberry-Atg4B^C74A^, followed by selection with medium containing 400 µg/mL G418 sulfate (Thermo Fisher Scientific). Single colonies of expressing cells were then isolated and expanded.

### siRNAs and plasmids

We used the following siRNAs from Sigma-Aldrich: Rap1A#1 (SASI_Hs1_00040405), Rap1A#2 (SASI_Hs2_00305929), Rap1B#1 (SASI_Hs2_00305940), Rap1B#2 (SASI_Hs2_00305936), TSC2 (SASI_Hs02_00312104) and universal negative control siRNA (SIC001).

EGFP-Rab7WT, EGFP-Rab7Q67L, and EGFP-Rab7T22N plasmids were a kind gift from John Blenis lab. pCDH-puro-myr-HA-Akt1 was a gift from Jialiang Wang (Addgene plasmid # 46969) [34]. pmStrawberry-Atg4B^C74A^ was a gift from Tamotsu Yoshimori (Addgene plasmid # 21076).

### Reagents

Concanamycin A (Abcam) was used at a concentration of 1 µM. Chloroquine (Sigma-Aldrich) was used at final concentrations of 20 µM or 40 µM. GDC0941 and GSK690693 (both from Selleck Chemical) were used at 500 nM and 10 µM, respectively.

### Antibodies

Western Blot. The following antibodies were purchased from Cell Signaling Technology: p-S6K1 T389 (9234), S6K1 (2708), p-4E-BP1 Ser65 (9451), 4E-BP1 (9644), mTOR (2983), p-Akt S473 (4060), Rap1A + B (2399), Akt1 (2938), TSC2 (3302), Rab7 (2094). Other antibodies included: GCN2 (Novus Biologicals, MAB6878), LC3B (Novus, NB100-2220), β-actin (Santa Cruz, sc-47778), p-GCN2 T899 (Abcam, ab75836). All primary antibodies were used at a 1:1000 dilution and secondary antibodies conjugated to horseradish peroxidase (GE Healthcare) or IRDye® fluorophores (LI-COR Biosciences) were used at a 1:5000 dilution in EveryBlot Blocking Buffer (Bio-Rad Laboratories).

Immunofluorescence. The following primary antibodies were used at a 1:100 dilution: LC3B (Cell Signaling Technology, 3868), LAMP2 (Santa Cruz, sc-18822), mTOR (Cell Signaling Technology, 2983S). Secondary antibodies conjugated to Alexa Fluor dyes (Alexa Fluor 488, 568, and 647; Thermo Fisher Scientific) were used at a 1:1000 dilution. Phalloidin was used at a 1:400 dilution for identifying cell ROIs (Thermo Fisher Scientific, A22287).

### Transfection

siRNA and DNA transfections were carried out using Lipofectamine 2000 (Thermo Fisher Scientific) according to the manufacturer’s protocol. siRNA transfection was performed with 100 pmol siRNA per 6-well and 20 pmol siRNA per 24-well. U2OS cells were transfected with Rap1 siRNA twice on consecutive days prior to starvation. DNA transfection for HEK293A cells was performed with 1.5 µg DNA per 6-well and 0.3 µg DNA per 24-well. For U2OS cells, 0.4 µg DNA per 24-well was used. Prior to seeding, plates were coated with 10 µg/mL laminin (Sigma-Aldrich) in PBS for a minimum of 4 h at 37 °C.

### Cell lysis and western blot

Cells were washed once with cold PBS and lysed on ice using RIPA buffer containing cOmplete protease inhibitors and PhosSTOP phosphatase inhibitors (both from Roche). Cell lysates were run on Any kD Mini-PROTEAN TGX Stain-Free precast gels (Bio-Rad Laboratories), and transferred to either nitrocellulose or PVDF membranes, which were then blocked using EveryBlot Blocking Buffer (Bio-Rad Laboratories). Due to technical reasons, immunoblots of phosphorylated proteins and their respective total proteins often had to be processed on separate blots. Membranes were incubated in primary antibody solution at 4 °C overnight, followed by secondary antibody incubation at room temperature for one hour. For chemiluminescence detection, membranes were developed in SuperSignal West Pico PLUS Chemiluminescent Substrate or SuperSignal West Femto Maximum Sensitivity Substrate (Thermo Fisher Scientific) and captured using an Amersham Imager 680 system (GE Healthcare) or an Odyssey Fc Imager (LI-COR). For multiplex fluorescence blotting, membranes were incubated in IRDye 680RD or 800CW (LI-COR) and imaged with an Odyssey Fc Imager (LI-COR).

### Immunofluorescence

HEK293A cells were seeded at 15,000–30,000 cells on glass coverslips coated with Poly-L-ornithine (Sigma-Aldrich) and laminin. Cells were fixed in 4% formaldehyde for 15 min at room temperature followed by three washes with PBS and permeabilization with 0.2% Triton X-100 for 10 min at room temperature. Samples were blocked for 1 h in 1X Carbo-free Blocking Solution (Vector Laboratories), followed by overnight incubation with primary antibodies in 1:100 dilution at 4 °C. Thereafter, samples were washed three times with PBS and incubated for 1 h with corresponding secondary antibodies plus DAPI (Thermo Fisher) in 1:1000 dilution. This was followed by three PBS washes (10 min each) and mounting of the coverslips on glass slides using Dako Fluorescence Mounting Medium (Agilent Technology).

Fluorescent images were captured on a Z1/7 (Zeiss) widefield microscope using 40× or 63× objectives at room temperature and the ZEN software (Zeiss). Confocal images were captured with a Zeiss LSM700 confocal microscope using a 40× or 63×-oil objective at room temperature and the ZEN software, or a Nikon A1R+ confocal microscope using the 63×-water objective in room temperature and the NIS-elements software. Appropriate bandpasses for fluorescence detection were used together with 1–6× scanner zoom. The microscopic fields imaged were randomly selected. Images were always captured using identical settings across the experiments. Representative images are shown at the same magnification and using identical acquisition and display settings.

### Image analysis

**Fig. 1:**
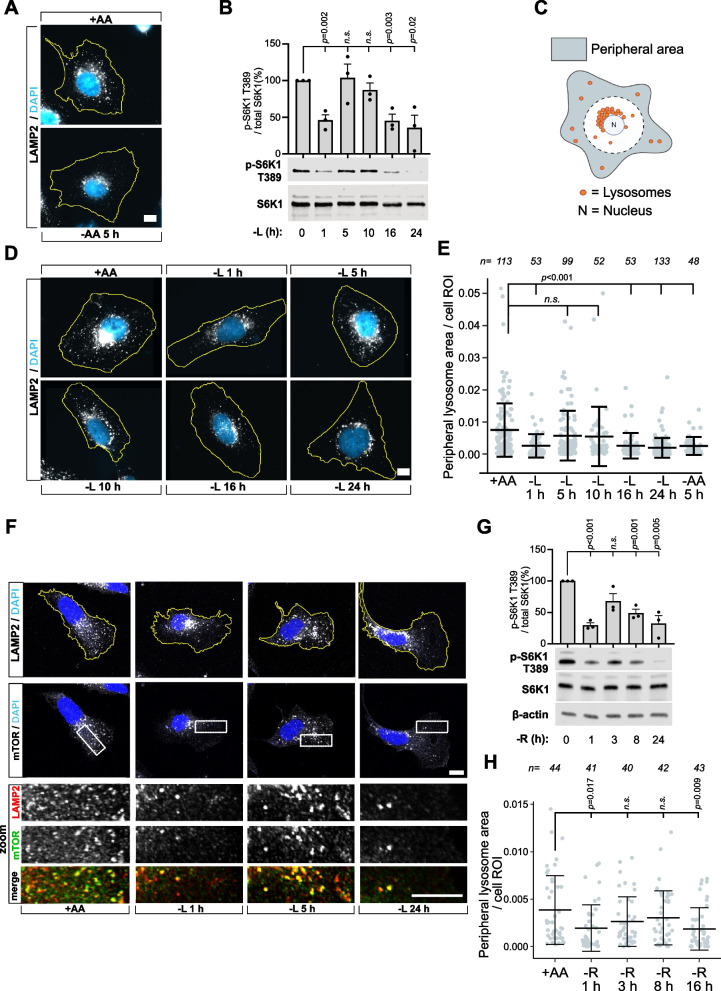
Sustained mTORC1 inhibition by leucine starvation involves the translocation of peripheral lysosomes to the perinuclear region. **A** Representative immunofluorescence images of endogenous LAMP2-marked lysosomes in U2OS cells starved of all amino acids (-AA) for 5 h. Cell boundaries are outlined in yellow; nuclei are stained with DAPI (blue). **B-F** U2OS cells were starved of leucine (-L) for the indicated timepoints, after which mTORC1-associated signaling was evaluated by immunoblotting for phosphorylated S6K1 T389 (**B**), and the change in peripheral lysosome localization was quantified in an automated fashion as described in Materials and Methods (**C**-**E**). Schematic showing the peripheral region (grey), defined as the area extending 10 μm from the nucleus, used for quantification of lysosome positioning is shown in (**C**). Also see Figure S1B. Representative immunofluorescence images of LAMP2-marked lysosomes at the indicated leucine starvation time points are shown in (**D**), with quantification of peripheral lysosome localization from (**A**) and (**D**) shown in (**E**). **F** Representative single confocal z-slices of mTOR localization to LAMP2-marked lysosomes across the indicated leucine starvation timepoints in U2OS cells. Cell boundaries are outlined in yellow, and the area magnified in the lower images is indicated by a white box. See Fig S1E for quantifications. **G, H** U2OS cells were starved with arginine (-R) for the indicated timepoints and analyzed for mTORC1 signaling (**G**) and lysosome positioning (**H**). See Fig S1F for images. Scale bars = 10 μm. Dialyzed FBS (dFBS) was included in all amino acid starvation experiments. In (**E, H**), "n" denotes the number of individual cells analyzed across three independent experiments. Immunofluorescence image quantifications are presented as mean values ± s.d.; one-way ANOVA with Tukey’s post hoc test. Immunoblot quantifications across at least three individual experiments are presented as mean ± SEM and were statistically analyzed using two-tailed unpaired Student’s t-test (*n.s.,* not significant; *p* ≥ 0.05). In (**H**), outliers were detected and removed using the 1.5× Interquartile Range (IQR) method before statistical testing

**Fig. 2:**
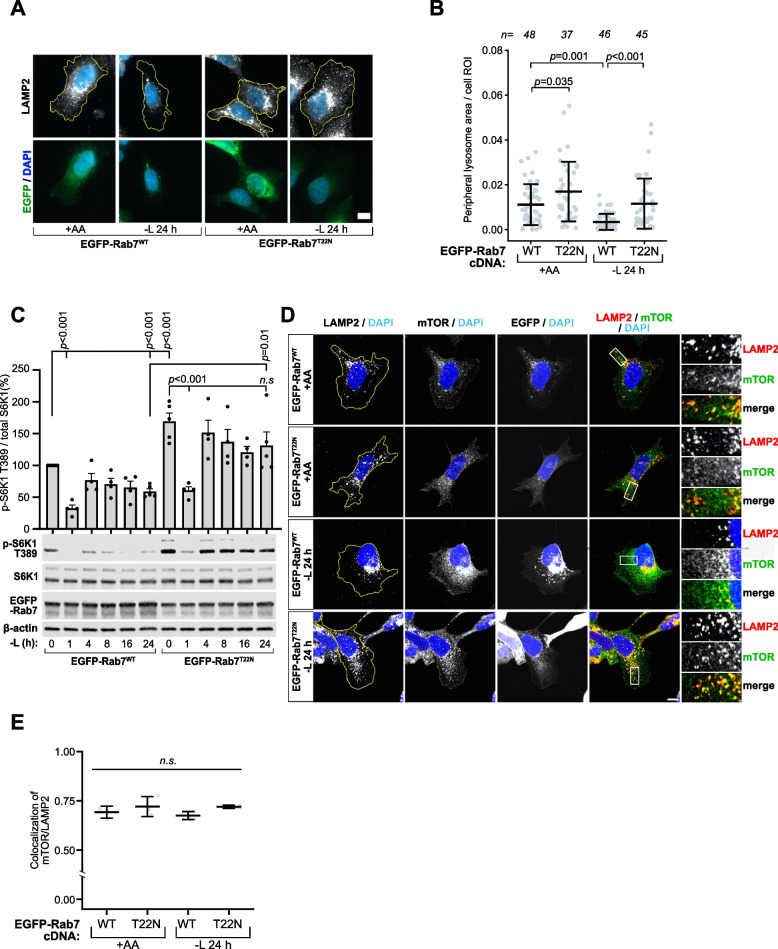
Rab7 signaling is required for peripheral lysosome relocation and sustained mTORC1 suppression during prolonged leucine starvation. **A, B** Immunofluorescence images showing lysosome distribution in U2OS cells expressing wild-type EGFP-Rab7 (EGFP-Rab7^WT^) or dominant-negative EGFP-Rab7 T22N (EGFP-Rab7^T22N^) under amino acid-fed (+ AA) or 24 h leucine-starved (− L) conditions. Peripheral lysosome localization is quantified in (**B**). **C** mTORC1 signaling was assessed by immunoblot analysis of lysates from HEK293A cells expressing the indicated EGFP-Rab7 constructs and subjected to leucine starvation for up to 24 h. (**D-E**) Representative confocal images from a single focal plane showing LAMP2-marked lysosomes (red) and mTOR (green) in leucine-starved HEK293A cells (**D**), with mTOR/LAMP2 colocalization quantification in (**E**) from three independent biological replicates (n = 3). All experiments were repeated at least three times. Scale bars (**A**,** D**) = 10 μm. dFBS was included in all leucine starvation experiments. In (**B**), "n" denotes the number of individual cells analyzed across 3 independent experiments. Immunofluorescence quantifications are presented as mean values ± s.d.; one-way ANOVA with Tukey’s post hoc test. Immunoblot quantifications across at least three individual experiments are presented as mean ± SEM and were statistically analyzed using two-tailed unpaired Student’s t-test (*n.s.,* not significant; *p* ≥ 0.05). In (**B**), outliers were detected and removed using the 1.5× Interquartile Range (IQR) method before statistical testing

**Fig. 3:**
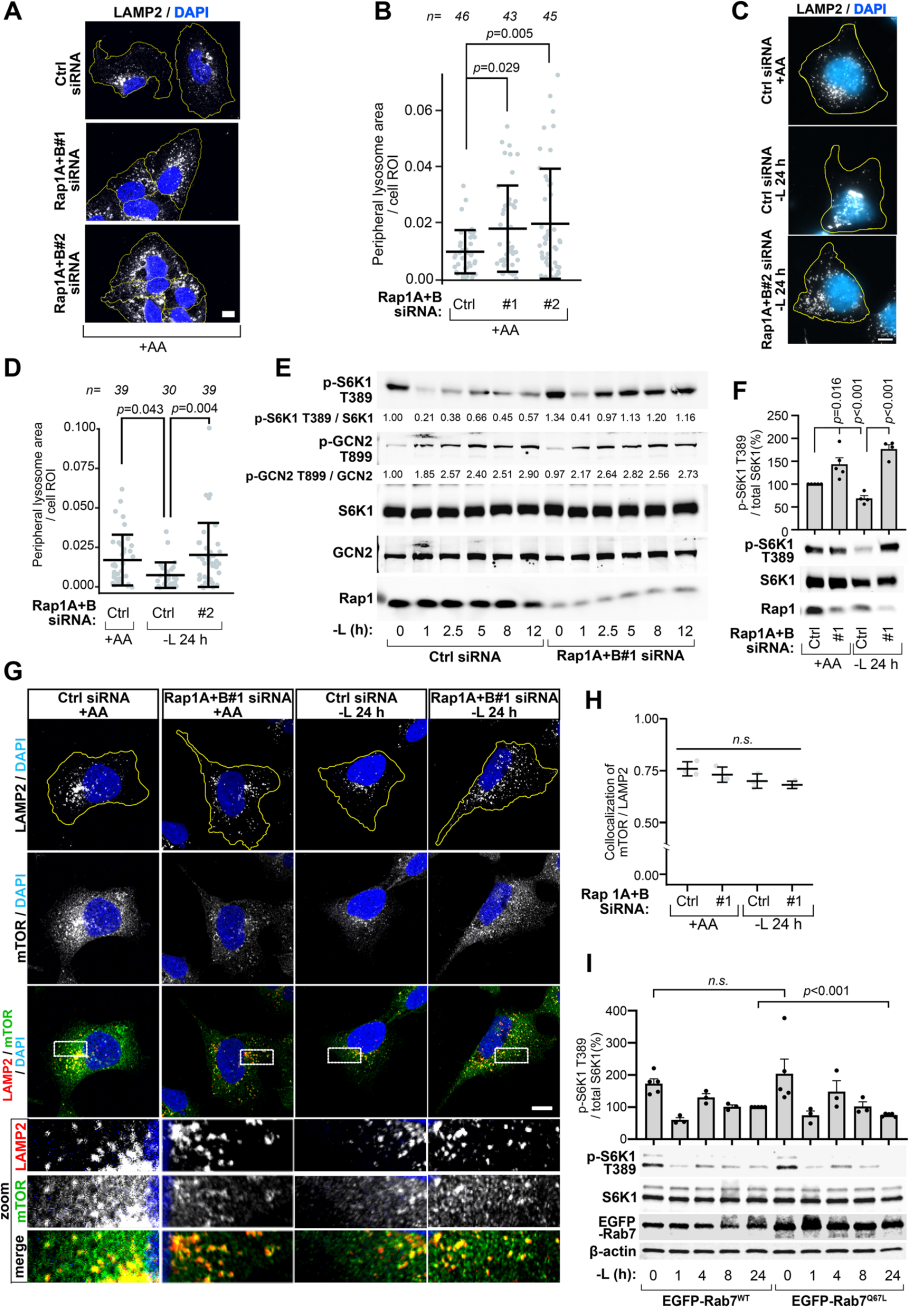
Rap1 activity is required for peripheral lysosome relocation and sustained mTORC1 suppression during prolonged leucine starvation. **A–D** Effects of Rap1 depletion on lysosome organization under amino acid-fed and prolonged leucine deprivation. U2OS (**A**,** B**) or HEK293A (**C**,** D**) cells were transfected with control or Rap1A + B siRNA and cultured in amino acid-fed (**A**,** B**) or leucine-free (**C**,** D**) medium**.** Representative z-stack confocal maximum-intensity projections (**A**) and fluorescence images (**C**) are shown, with quantifications of lysosome peripheral distribution in (**B**,** D**). **E, F** Immunoblot analysis of mTORC1-associated signaling in Rap1-depleted HEK293A cells starved for the indicated time points. **G** Representative single confocal z-slices of mTOR and LAMP2-marked lysosomes in HEK293A cells transfected with Rap1A + B siRNA under amino acid-fed or leucine-starved conditions, with quantifications of mTOR/LAMP2 colocalization in (**H**). **I** Immunoblot analysis of mTORC1 signaling in HEK293A cells expressing EGFP-Rab7^WT^ or EGFP-Rab7^Q67L^. All experiments were repeated at least three times. Scale bars: (**A**,** C**,** G**) = 10 μm. dFBS was included in all leucine starvation experiments. In (**B**,** D**), "n" denotes the number of individual cells analyzed across 3 independent experiments, data are presented as mean values ± s.d.; with statistical analysis performed using one-way ANOVA with Tukey’s post hoc test. Immunoblot quantifications across at least three individual experiments are presented as mean ± SEM and were statistically analyzed using two-tailed unpaired Student’s t-test (*n.s.,* not significant; *p* ≥ 0.05). In (**B**), outliers were detected and removed using the 1.5× Interquartile Range (IQR) method before statistical testing

For quantification of lysosome peripheral localization, fluorescent images of randomly selected, sparsely seeded cells were captured as described above. The lysosomal peripheral distribution was quantified automatically using ImageJ/Fiji. Briefly, binary images of the lysosomes, nucleus and cell body (cell ROI) were generated for individual cells using thresholding settings appropriate for control samples and these settings were consistently applied throughout each experiment. The peripheral area was defined as the region extending more than 10 μm from the nucleus. The area of lysosomes located within this peripheral region was normalized either to the total cell area or to the total lysosomal area, as indicated in the figures. Example images of this process are shown in Fig. S1B. This quantification was repeated for multiple individual cells across three independent experiments. Data in Figs. 1A, D, 2A, 3C, S1F, S2A, S3C, S3E, S3G, and S4C fluorescent images were captured with a Z1/7 Zeiss widefield microscope. Data presented in Fig. 3A, B were obtained from a previous dataset of confocal images of U2OS cells that were reanalyzed [26]. Outliers were detected and removed using the 1.5× Interquartile Range (IQR) method before statistical testing in Fig. 1H, 2B, 3B and S2B.

For colocalization analysis, Pearson’s correlation coefficients were quantified between mTOR and LAMP2 signals within segmented cells using CellProfiler (v4.2.6). For each condition, 5–8 confocal images captured with the 40× objective were analyzed per condition across three independent biological replicates. Statistical analysis and data visualization were performed in R (version 4.4.1). For quantification of LC3B/LAMP2 overlaps and change in total LAMP2 area, confocal images of sparsely seeded HEK293A cells were captured using the 63× objective, as described above. Binary images of lysosomes and LC3B-autophagosomes were generated in ImageJ/Fiji from a maximum projection of five z-slices with 0.45 µm thickness. Thresholding settings optimized for control samples were consistently applied across each experiment. The LC3B/LAMP2 double-positive area was quantified relative to the total LC3B area in an automated manner across multiple images, each containing multiple cells. LAMP2 area was quantified relative to the total cell ROI in an automated manner across multiple images, each containing multiple cells.

Vesicle mean speed was analyzed using the TrackMate plugin (v3.4.0) for Fiji/ImageJ, using the DoG detector. Vesicle diameter was set to 1 µm with the appropriate settings and thresholds for control samples. An expanded dataset was used, incorporating additional cells from a previously collected dataset [26].

## Statistical analysis

All experiments were repeated at least three times unless noted in the legend. p-values were calculated using two-tailed unpaired Student's t-test or ANOVA with Tukey’s post hoc test, as indicated in figure legends. As mentioned above, outliers were detected and removed using the 1.5× Interquartile Range (IQR) method before statistical testing in Figs. 1H, 2B, 3B and S2B.

## Results

### Peripheral lysosome translocation to the perinuclear region is required for sustained mTORC1 suppression during leucine or arginine starvation

**Fig. 4:**
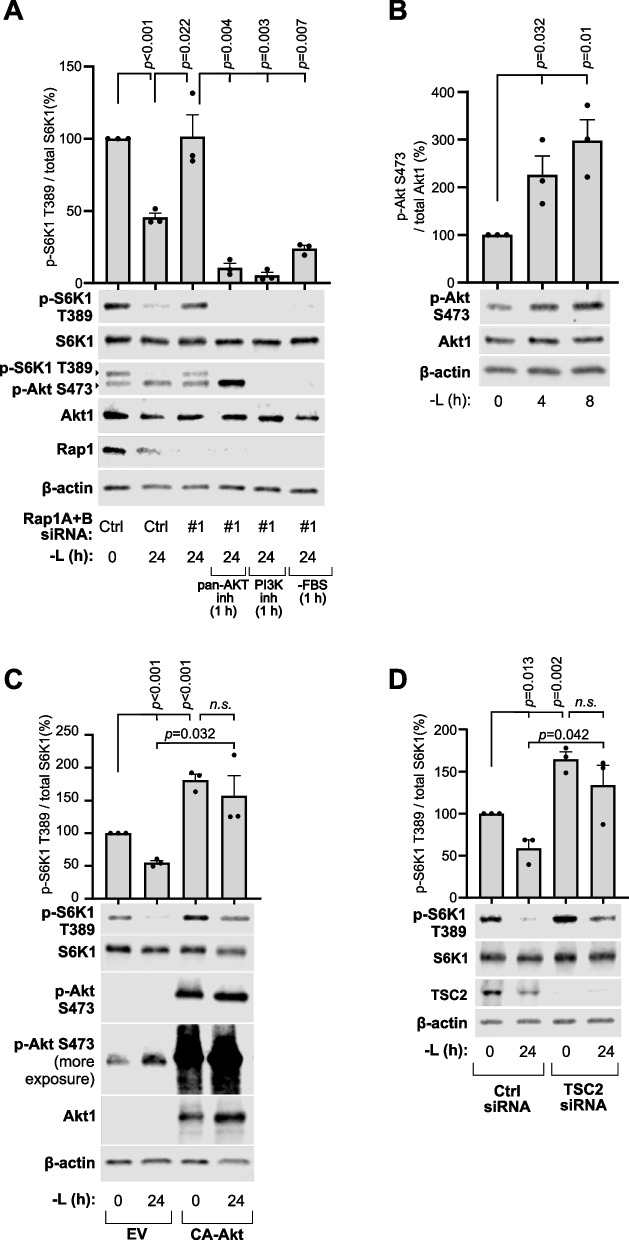
Sustained mTORC1 activity by elevated lysosome levels is dependent on PI3K/Akt growth factor signaling. **A** HEK293A cells depleted of Rap1A + B by indicated siRNA were leucine-starved for 24 h and treated during the final hour with either 10 μM pan-Akt inhibitor (GSK690693), 500 nM PI3K inhibitor (GDC0941), or FBS removal (-FBS), then analyzed by immunoblotting for the indicated proteins. **B** The phosphorylation status of Akt in HEK293A cells starved of leucine for the indicated time points. **C** mTORC1 signaling was assessed in HEK293A cells transfected with either empty vector (EV) or constitutively active myristoylated Akt cDNA (CA-Akt) leucine starvation for 24 h. **D** mTORC1 signaling was assessed in HEK293A cells transfected with either control siRNA or TSC2 siRNA prior to 24 h leucine starvation. dFBS was included in all leucine starvation experiments, except during the FBS-starvation assay in (**A**). Immunoblot quantifications across at least three individual experiments are presented as mean ± SEM and were statistically analyzed using two-tailed unpaired Student’s t-test (*n.s.,* not significant; *p* ≥ 0.05)

Complete amino acid deprivation induces a pronounced spatial reorganization of the lysosome system within the cytoplasm, characterized by the translocation of peripheral lysosomes to the perinuclear area within five hours of the onset of starvation (Fig. 1A) [23, 26] and dissociation of mTOR from lysosomes (Fig. S1A) [35]. Whether the relocation of peripheral lysosomes away from the plasma membrane, where growth factor signaling components are enriched, contributes to mTORC1 suppression remains unclear. Therefore, we focused on leucine starvation, which leads to delayed mTORC1 inactivation and continued association of mTOR with lysosomes [23, 29, 36, 37]. Accordingly, we starved U2OS cells of leucine for one to 24 h, maintaining dialyzed serum to preserve growth factor signaling, while quantifying changes in peripheral lysosome levels (see Materials and Methods and Fig. S1B) and assessing mTORC1 activity by measuring phosphorylation of its targets S6K1 at T389 (Fig. 1B) and 4E-BP1 at S65 (Fig. S1C). As reported previously [36, 37], leucine starvation led to acute but transient repression of mTORC1 signaling levels within one hour, followed by a brief recovery period during which activity returned to levels comparable to fed cells. Importantly, RagA/B GTPase activity was essential for mTORC1’s ability to rapidly inactivate in response to starvation, as no reduction in mTORC1 signaling was observed in starved RagA/B-deficient cells (Fig. S1D), consistent with their crucial role in signaling leucine to mTORC1 [12, 38]. After 10 h, mTORC1 signaling began to decrease again, reaching sustained repression by 24 h (Fig. 1B).

Interestingly, peripheral lysosome levels closely mirrored mTORC1 signaling output throughout the starvation time course (Fig. 1C-E). At the acute phase of starvation, peripheral lysosomes briefly accumulated in the perinuclear region, leading to reduced peripheral lysosome levels within one hour, which recovered by five hours as lysosomes regained a scattered distribution comparable to fed cells. After 10 h, when sustained mTORC1 repression was initiated, peripheral lysosomes permanently concentrated in the perinuclear region, resulting in significantly reduced peripheral lysosome levels by 24 h, comparable to complete amino acid starvation (Fig. 1A, D, E). Notably, mTOR association with lysosomes was observed throughout the starvation period, indicating mTORC1 signaling output may be modulated by lysosome positioning (Fig. 1F, Fig. S1E). We next tested whether a similar spatiotemporal relationship between mTORC1 signaling and lysosome positioning occurs during prolonged arginine starvation. Interestingly, arginine deprivation largely mirrored the effects of leucine starvation, exhibiting coordinated reductions in mTORC1 signaling and peripheral lysosome abundance during both acute and prolonged starvation (Fig. 1G-H; Fig. S1F), suggesting that the effect is not restricted to leucine.

To investigate whether peripheral lysosome translocation to the perinuclear region suppresses mTORC1 during leucine starvation, we disrupted Rab7 activity – a GTPase crucial for lysosome retrograde trafficking towards the nucleus along microtubules – through expression of dominant negative Rab7-T22N (Rab7^T22N^) [39–41]. Blocking retrograde trafficking robustly increased peripheral lysosome levels under fed conditions compared to Rab7 wild-type (Rab7^WT^) expression. This increase persisted at both one and 24 h of starvation (Fig. 2A-B, Fig. S2A-B), suggesting that Rab7 activity is required for starvation-induced retrograde lysosome transport. While Rab7^T22N^−expressing cells displayed elevated basal mTORC1 signaling under fed conditions, consistent with enhanced signaling from an expanded peripheral lysosome pool [23], a transient reduction in signaling was observed after one hour of leucine starvation, akin to control cells (Fig. 2C), implying that acute mTORC1 suppression occurs independently of lysosome spatial organization via Rag GTPase signaling. In contrast, mTORC1 signaling displayed a striking inability to downregulate during prolonged starvation, remaining hyperactivated at levels equivalent to the leucine-stimulated state (Fig. 2C, Fig. S2C), indicating that retention of peripheral lysosomes by Rab7^T22N^ expression prevented mTORC1 inactivation. Confocal analysis confirmed mTOR association with peripheral lysosomes during starvation (Fig. 2D-E). Collectively, these data suggest that peripheral lysosome translocation to the perinuclear region is critical for long-term mTORC1 repression, but not short-term.

To validate these results in a different setting, we employed siRNA to silence Rap1A and Rap1B GTPases (collectively referred to as Rap1), which we recently identified as important mediators of juxtanuclear accumulation of lysosomes upon complete amino acid deprivation, as well as normal lysosome trafficking [26]. As observed previously [26], Rap1 depletion disrupted lysosome spatiotemporal organization in fed cells, increasing lysosomal motility (Fig. S3A-B) and abundance (Fig. 3A, Fig. S3C-D). This resulted in a significant increase in peripheral lysosome levels under both fed and starved conditions (Fig. 3A-D, Fig S3E-F). Intriguingly, the Rap1-mediated elevation in peripheral lysosome levels closely mirrored the effect of Rab7^T22N^ on mTORC1 signaling. While there was a transient repression in signaling at one hour of starvation, mTORC1 failed to inactivate by 24 h of leucine starvation, displaying hyperactivity comparable to the fed state (Fig. 3E-F, Fig. S3I). Consistent with our observations from Rab7^T22N^-expressing cells, mTOR association with peripheral lysosomes was detected during leucine starvation in Rap1-depleted cells (Fig. 3G-H). Equivalent resistance to prolonged arginine starvation was observed after Rap1-depletion (Fig. S3G-I). To further validate that expansion of the peripheral lysosome pool impairs mTORC1 inactivation, we increased peripheral lysosome translocation via Arl8b expression [19, 23], which resulted in elevated mTORC1 signaling after 24 h of leucine starvation compared to control cells (Fig. S3J). Taken together, these data corroborate that a reduction in peripheral lysosome levels is critical for sustained mTORC1 inactivation during leucine starvation.

We also examined whether changes in lysosome distribution affect general control nonderepressible 2 (GCN2) signaling, which is activated by leucine starvation and is important for sustained mTORC1 inhibition under these conditions [37, 42]. However, neither GCN2 T899 auto-phosphorylation (Fig. 3E) nor protein levels of its downstream translation target ATF4 (Fig. S4A) were altered in Rap1-depleted cells under leucine starvation, indicating that changes in peripheral lysosome levels do not influence mTORC1 through altered GCN2/ATF4 signaling.

Next, we decreased peripheral lysosome levels by boosting lysosomal retrograde trafficking using constitutively active Rab7-Q67L (Rab7^Q67L^) and assessed the impact on mTORC1 inactivation [39]. While acute suppression of mTORC1 was evident in Rab7^Q67L^ cells, mTORC1 activity was significantly further repressed at 24 h of starvation compared to Rab7^WT^ control cells (Fig. 3I), supporting the finding that peripheral lysosome localization determines long-term mTORC1 inactivation.

## Sustained mTORC1 activity by elevated lysosome levels is dependent on PI3K/Akt growth factor signaling

Lysosome positioning near the plasma membrane potentiates mTORC1 activation as a result of increased exposure to stimulatory Akt signals [22, 23]. We therefore asked whether Akt signaling similarly sustains mTORC1 activity under conditions of elevated peripheral lysosome levels during prolonged leucine starvation. To test this, we inhibited Akt signaling in Rap1-depleted cells with expanded peripheral lysosome pools using serum starvation (-FBS), the pan-Akt inhibitor GSK690693 (10 µM), or the PI3K inhibitor GDC0941 (500 nM), for 6 h (Fig. S4B) or 24 h of leucine starvation (Fig. 4A). Interestingly, all treatments robustly inactivated mTORC1 signaling at both starvation time points (Fig. 4A, Fig. S4B), demonstrating that continuous PI3K/Akt signaling is required for persistent mTORC1 activity mediated by increased peripheral lysosome localization. Notably, leucine starvation also elevated basal Akt signaling levels, as judged by increased Akt S473 phosphorylation (Fig. 4A-B), consistent with previous observations [36].

To test whether heightened growth factor signaling to mTORC1 impedes pathway deactivation during prolonged leucine starvation, we hyperactivated Akt signaling by expressing myristoylated Akt (constitutively active Akt; CA-Akt), which concentrates at the plasma membrane [43] without increasing peripheral lysosome levels (Fig. S4C-D). Akt hyperactivation enhanced basal mTORC1 signaling and markedly impaired its ability to undergo repression at 24 h of starvation (Fig. 4C), mimicking the effects of Rab7^T22N^ expression and Rap1 depletion (Fig. 2C, 3F). A similar impairment in mTORC1 inactivation was observed following siRNA-mediated knockdown of TSC2 (Fig. 4D). Together, these data demonstrate that intensified growth factor signaling to mTORC1 compromises the pathway’s ability to deactivate, supporting a model in which lysosome relocation away from these signals suppresses mTORC1 activity.

## Peripheral lysosomes maintain mTORC1 without relying on lysosome catabolic function and autophagy

**Fig. 5:**
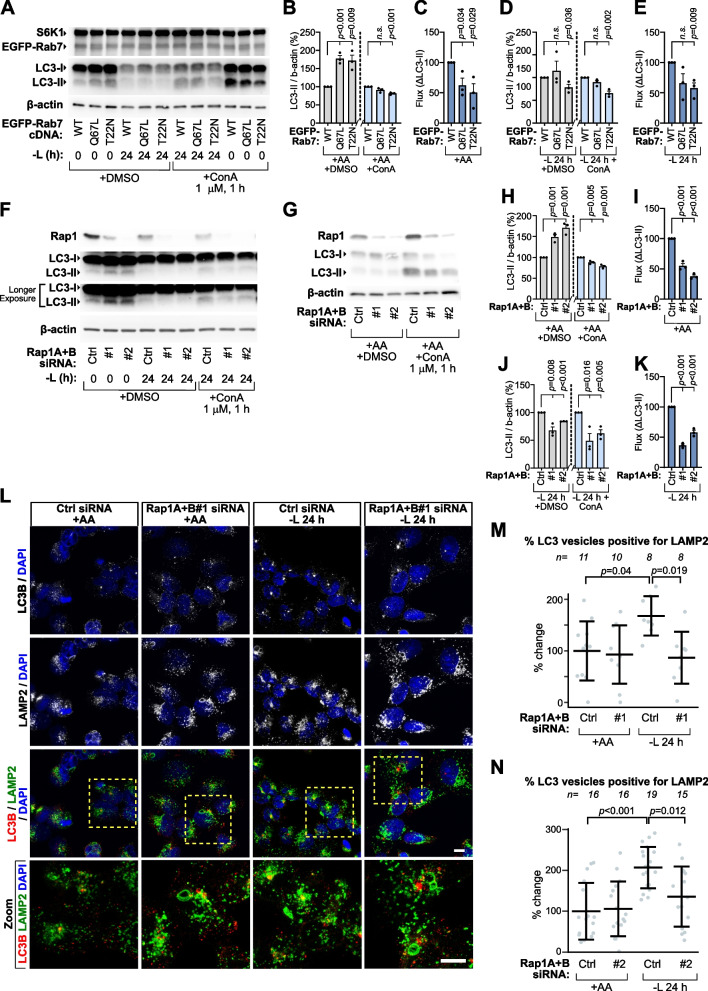
Autophagy is suppressed in cells with disrupted Rab7 and Rap1 signaling. **A-K** HEK293A cells expressing the indicated EGFP-Rab7 constructs (**A-E**) or depleted of Rap1A + B by siRNA (**F-K**) were cultured in complete medium or subjected to 24 h leucine starvation (-L) and immunoblotted for endogenous targets. Concanamycin A (ConA; 1 μM) was added for 1 h prior to lysis to block lysosomal degradation. Quantifications of LC3-II normalized to β-actin and to the corresponding control condition are shown for each treatment (**B**,** D**,** H**,** J**). Autophagic flux, estimated as the difference in normalized LC3-II between ConA-treated and untreated cells (ΔLC3-II), was calculated for each Rab7- and Rap1-perturbed conditions under fed and starved states, as indicated (**C**,** E**,** I**,** K**). **L-N** Representative z-stack confocal max-intensity projections of LAMP2-marked lysosomes and LC3B-marked autophagosomes in starved HEK293A cells transfected with Rap1A + B siRNA. The percentage of LC3-positive vesicles overlapping with LAMP2 was quantified in an automated fashion for Rap1A + B siRNA #1 (**M**) and #2 (**N**). dFBS was included in all leucine starvation experiments. All experiments were repeated three times. Scale bars: (**L**) = 10 μm. In (**M**,** N**), "n" denotes the number of individual images analyzed across three independent experiments with multiple cells in each image, data are presented as mean values ± s.d.; with statistical analysis performed using one-way ANOVA with Tukey’s post hoc test. Immunoblot quantifications across at least three individual experiments are presented as mean ± SEM and were statistically analyzed using two-tailed unpaired Student’s t-test (*n.s.*, not significant; *p* ≥ 0.05)

Given that our data suggested increased peripheral lysosome abundance sustains mTORC1 activity via PI3K/Akt signaling, we hypothesized that this effect is independent of autophagy, which can reactivate mTORC1 during starvation through nutrient recycling [44]. This is consistent with earlier findings that peripheral expansion of the lysosome pool suppresses autophagy by disrupting lysosome maturation and autophagosome–lysosome fusion [15, 18, 23, 39, 45]. To test whether autophagic flux is suppressed in Rab7^T22N^ cells, we assessed changes in LC3B-II levels in conjunction with the inhibition of lysosome catabolic function using the v-ATPase inhibitor ConA (1 µM, 1 h). Under nutrient-replete conditions, Rab7^T22N^ cells displayed elevated LC3B-II levels compared to Rab7^WT^ controls, whereas upon ConA treatment, LC3B-II levels were reduced in Rab7^T22N^ cells relative to Rab7^WT^ (Fig. 5A-B). Quantification of LC3B-II flux—calculated as the difference in LC3B-II levels in the presence versus absence of ConA—revealed a marked reduction in Rab7^T22N^ cells relative to Rab7^WT^, indicating impaired autophagic flux (Fig. 5C) [46]. Reduced flux was also noted for Rab7^Q67L^ cells (Fig. 5B-C). During leucine starvation, LC3B-II levels failed to accumulate in Rab7^T22N^ cells and were further reduced upon ConA treatment (Fig. 5D), resulting in a significant decrease in LC3B-II flux (Fig. 5E), with no significant change observed in Rab7^Q67L^ cells relative to Rab7^WT^ (Fig. 5D-E). The effect of Rap1 depletion on LC3B-II levels and flux closely mirrored that of Rab7^T22N^ expression, implying reduced autophagic flux (Fig. 5F-K), which had not been observed after one hour of complete amino acid starvation [26]. Further supporting the requirement of Rap1 for optimal autophagic flux, Rap1 depletion led to reduced overlap between LC3B-marked autophagosomes and LAMP2-lysosomes under leucine starvation (Fig. 5L-N), indicative of a reduction in lysosome-autophagosome encounters.

**Fig. 6:**
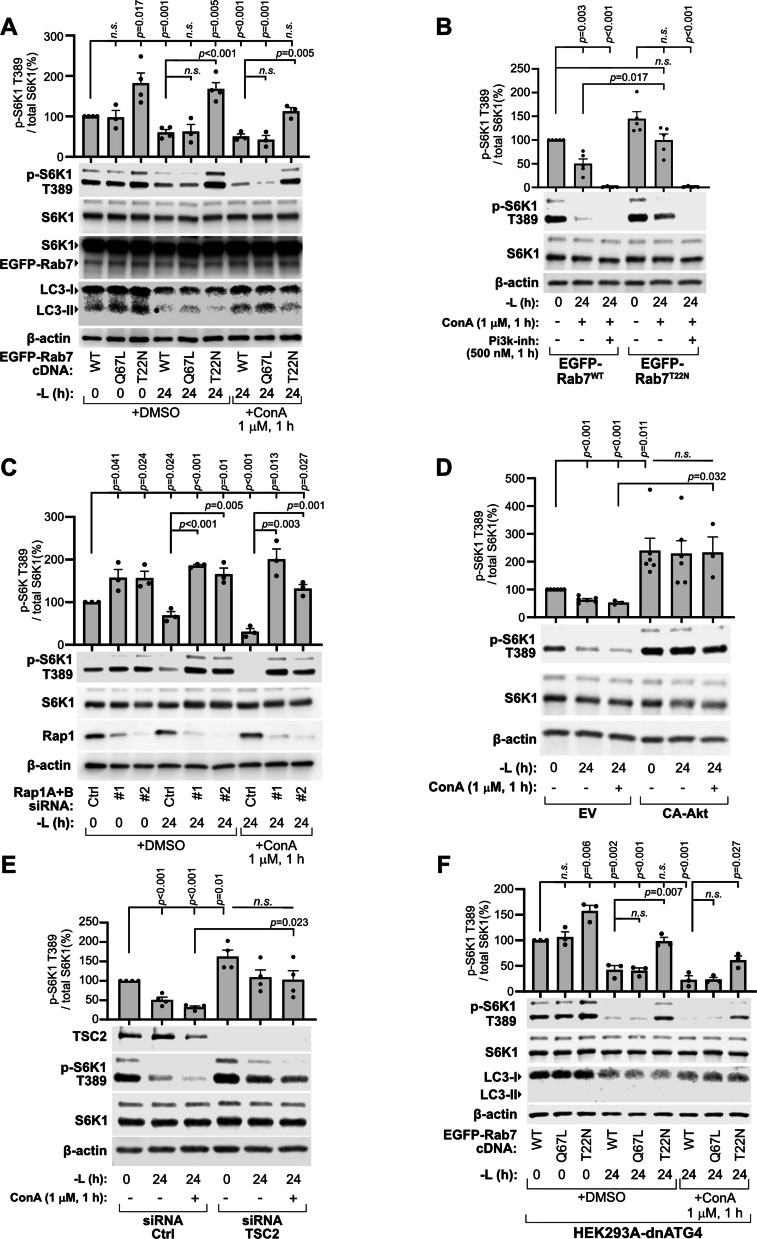
The maintenance of mTORC1 by peripheral lysosomes depends on PI3K/Akt signaling and is not driven by lysosomal catabolic processes. **A–E** HEK293A cells were transfected as indicated, starved of leucine for 24 h (-L), treated with concanamycin A (ConA; 1 μM) for 1 h prior to lysis and analyzed for mTORC1 signaling by immunoblotting. In (**A**), lysates corresponding to Fig. 5A were reprobed for the indicated proteins. In (**B**), cells were treated with 500 nM PI3K inhibitor (GDC0941) for one hour prior to lysis in the indicated conditions. EV = empty vector. **F** Analysis of mTORC1 signaling in macroautophagy-deficient HEK293A cells stably expressing dn-ATG4 and transiently expressing the indicated EGFP-Rab7 constructs. dFBS was included in all leucine starvation experiments. Immunoblot quantifications across at least three individual experiments are presented as mean ± SEM and were statistically analyzed using two-tailed unpaired Student’s t-test (*n.s.,* not significant; *p* ≥ 0.05)

**Fig. 7:**
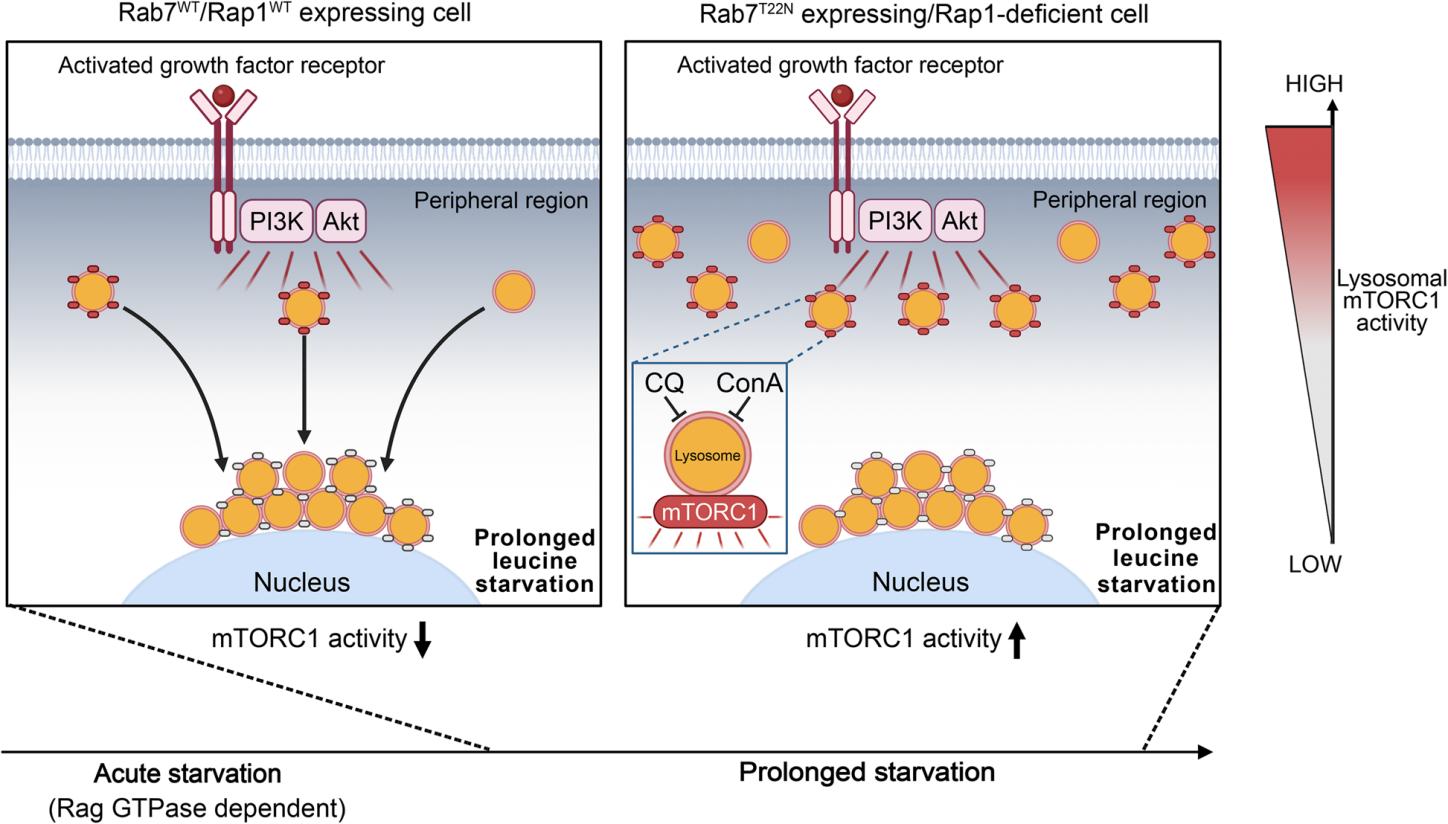
Model. Left box of the model: During prolonged leucine or arginine starvation, sustained mTORC1 inactivation requires that peripheral lysosomes translocate away from the plasma membrane and accumulate in the perinuclear region. Because a pool of mTORC1 remains active on lysosomes under starvation, distancing lysosomes from the plasma membrane—where PI3K/Akt signals are concentrated—reduces PI3K/Akt input to the pathway, which is necessary for sustained mTORC1 suppression. Starvation-induced lysosome reorganization depends on Rab7 and Rap1 signaling. Right box of the model: In Rab7^T22N^- or Rap1-depleted cells with an expanded peripheral lysosome pool, mTORC1 remains active during prolonged starvation due to increased exposure to stimulatory PI3K/Akt signals, which prevents sustained pathway inactivation. This persistent mTORC1 activity is not driven by amino acid regeneration from canonical macroautophagy, as it remains even when lysosomal catabolic function is disrupted by ConA or CQ

Supporting the notion that increased peripheral lysosome levels sustain mTORC1 signaling independently of lysosomal catabolic function and autophagy, mTORC1 signaling remained elevated under ConA treatment in leucine-starved Rab7^T22N^-expressing cells, at levels comparable to Rab7^WT^ in nutrient-replete conditions, in sharp contrast to Rab7^WT^ or Rab7^Q67L^ cells (Fig. 6A). This resistance was abolished by PI3K inhibition (Fig. 6B). Likewise, mTORC1 signaling remained insensitive to ConA treatment in leucine-starved Rap1-depleted cells (Fig. 6C). Hyperactivating growth factor input to mTORC1 by expression of CA-Akt (Fig. 6D) or TSC2 depletion (Fig. 6E) similarly resulted in sustained mTORC1 signaling during starvation, despite inhibition of lysosome catabolic function by ConA. To further explore whether elevated peripheral lysosome levels support mTORC1 signaling independently of autophagy, we impaired lysosome catabolic function continuously by CQ (40 µM) during the final six hours of the 24-h leucine starvation. Also under these conditions, mTORC1 signaling was sustained by peripheral lysosome expansion through Rab7^T22N^-expression (Fig. S4E). Even when lysosome function was targeted with CQ (20 µM) during the entire 24-h starvation, mTORC1 signaling remained elevated in Rap1-depleted cells (Fig. S4F). We next generated autophagy-deficient cells by stably expressing Atg4B^C74A^, which blocks autophagosome biogenesis (resulting in a complete absence of LC3-II; Fig. 6F) [47], and found that mTORC1 signaling remained significantly elevated in Rab7^T22N^−expressing cells relative to Rab7^WT^ controls under prolonged leucine starvation plus ConA. In conclusion, we propose that peripheral lysosome levels, by impacting PI3K/Akt signaling input to the pathway, determine the ability of mTORC1 to inactivate during prolonged leucine starvation, independently of autophagy (Fig. 7).

## Discussion

While the peripheral lysosomal pool has been suggested to dominate mTORC1 pathway activation [23], whether changes in its pool size affect mTORC1 inactivation has remained unclear. Here, we demonstrate for the first time that peripheral lysosome translocation to the perinuclear region is required for sustained mTORC1 repression in response to leucine or arginine starvation. Importantly, we also find that when the peripheral lysosome pool is aberrantly increased, lysosomal catabolic function can be severely compromised by pharmacological inhibition during starvation without abolishing mTORC1 activity, arguing that the increased resistance of mTORC1 is largely independent of lysosomal catabolism and autophagy, and instead mediated by the peripheral lysosome pool even when catabolically impaired. In agreement with the established role of Rag GTPases in signaling leucine to mTORC1 [11–13], leucine or arginine starvation acutely suppresses mTORC1 in a Rag-GTPase-dependent manner. However, this response is only partial, as PI3K/Akt growth factor signaling preserves residual mTORC1 activity during later stages of starvation, suggesting that lysosome translocation away from the plasma membrane reduces these stimulatory inputs and is critical for sustained pathway inactivation. Consistent with this model, increasing peripheral lysosome levels sustains mTORC1 signaling under prolonged starvation in a manner dependent on continuous PI3K/Akt signaling.

Peripheral lysosome relocation to the perinuclear region occurs during complete amino acid deprivation [23, 25–27]; however, under these conditions, mTORC1 signaling is inactivated independently of the organization of the lysosome network, as mTORC1 dissociates from lysosomes [35]. In contrast, our data indicates that a pool of mTOR remains associated with and continuously activated at lysosomes during leucine starvation, as reported previously under milder starvation protocols [23, 29], rendering the pathway sensitive to spatial changes in lysosome organization during prolonged starvation and growth factor stimulation. However, peripheral lysosome levels do not influence acute mTORC1 suppression, consistent with multiple independent mechanisms controlling mTORC1 signaling [37, 48–50], including Rag-GTPases and their associated components [4, 11, 13, 38, 51, 52], mTORC1 lysosomal autoregulation, where mTORC1 activity itself controls release from lysosomes [53, 54], as well as non-lysosomal regulation [55, 56]. This complex temporal dimension to mTORC1’s lysosomal regulation warrants further investigation.

Our data support the established model that the proximity of lysosomes to growth factor signaling components concentrated at the plasma membrane is a major determinant of overall mTORC1 signaling output, by modulating the magnitude of growth factor signaling input to the pathway [21–23, 57]. Previously, this model has exclusively been studied in the context of mTORC1 activation [21–23], with our work expanding it to the dynamics of mTORC1 inactivation. While the model is based on the generally accepted assumption that reducing the distance between intracellular pathway components enhances the kinetics and magnitude of signaling, it remains to be determined whether additional factors influenced by lysosome positioning contribute to mTORC1 inactivation during prolonged starvation. Such factors could localize to plasma-membrane bound focal adhesions, as recently proposed for growth factor signaling components [57]. Nonetheless, given that PI3K/Akt signaling is induced by both amino acid starvation and mTORC1 inactivation through multiple feedforward loops [36, 58–63], our model provides an explanation for why lysosome relocation away from the plasma membrane is required for sustained mTORC1 inactivation (See model in Fig. 7).

Our work reveals several intriguing questions that require further investigation. One is regarding the extent to which excess PI3K/Akt input to mTORC1 can compensate for insufficient amino acid signaling. Hyperactivated growth factor signaling has been shown to impair mTORC1 inactivation during amino acid starvation across multiple cellular contexts, including TSC2 deficiency [35, 64–66], Ras-MEK hyperactivation [67], and in various cancer cell lines [68, 69]. Thus, although growth factor signals are transmitted to mTORC1 separately from leucine sufficiency signals, there appear to be conditions under which increased input from one pathway overrides the lack of another. However, whether this poorly understood effect is specific to leucine and arginine starvation, or can be extended to other forms of starvation, remains to be determined.

Another pertinent question is what mechanisms drive lysosome translocation in response to acute and prolonged leucine starvation. While we find that the perinuclear lysosome clustering critically depends on Rab7 and Rap1 signaling, the upstream and downstream mediators of this response remain to be determined. A third intriguing question is whether other cellular or physiological contexts exist during which catabolically impaired lysosomes sustain mTORC1 signaling. In our study, we specifically examined mTORC1 resistance to CQ and v-ATPase inhibition during prolonged leucine starvation, however, under these conditions, v-ATPase inhibition is not necessarily expected to further suppress mTORC1 beyond the effects of leucine starvation, given its role in amino acid transmission within the same signaling axis [4]. Even so, considering that lysosome catabolic dysfunction is a hallmark of several human diseases, including lysosomal storage disorders where persistent mTORC1 has been reported [70], the dependence of lysosomal mTORC1 activity on catabolic function may have broader relevance than observed here. This includes the possibility that mTORC1, as a negative regulator of autophagy, may persist during starvation independently of autophagy-derived nutrient supply [5, 44].

Collectively, our findings highlight lysosomal spatial organization as a key determinant of mTORC1’s ability to inactivate under nutrient scarcity. Our results may have relevance for therapeutic approaches, particularly in strategies targeting specific amino acid depletion or lysosomal catabolism inhibition, as suggested for various cancer treatments [71].

## Supplementary Information

Supplementary Material 1.

## Data Availability

No datasets were generated or analysed during the current study.

## Abbreviations

CQ: Chloroquine
ConA: Concanamycin A
mTORC1: Mechanistic target of rapamycin complex 1
GCN2: General control nonderepressible 2
TSC: Tuberous sclerosis complex
