# Supplementary material for: Peripheral lysosome levels dictate mTORC1 inactivation even when catabolically impaired
Source: Cell Commun Signal. 2026 Jan 20;24:56. doi: 10.1186/s12964-026-02659-9 (PMC12849086; doi:10.1186/s12964-026-02659-9)

Fig 1B

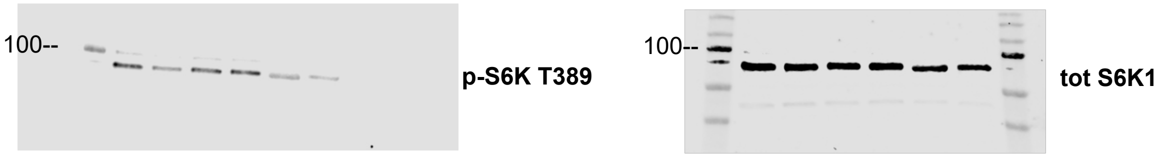
100--
p-S6K T389
100--
tot S6K1

Fig 1G

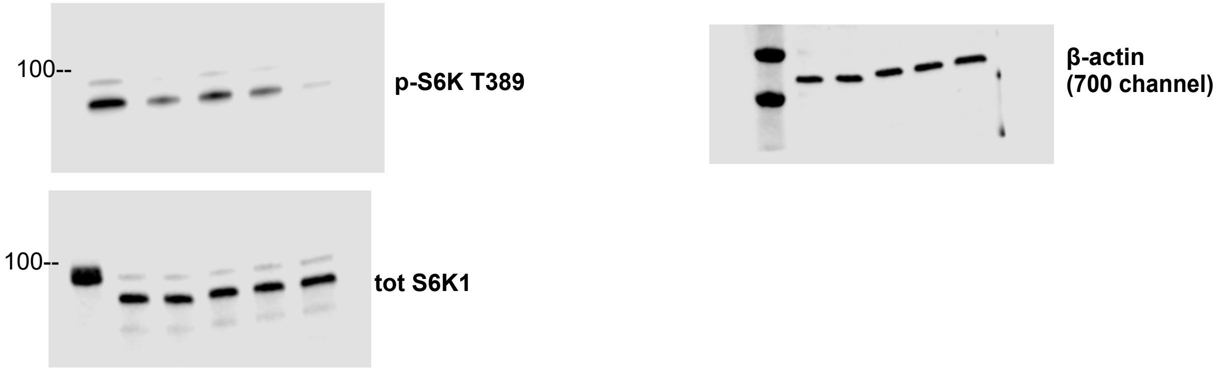
100--
p-S6K T389
100--
tot S6K1
β-actin
(700 channel)

Fig 2c

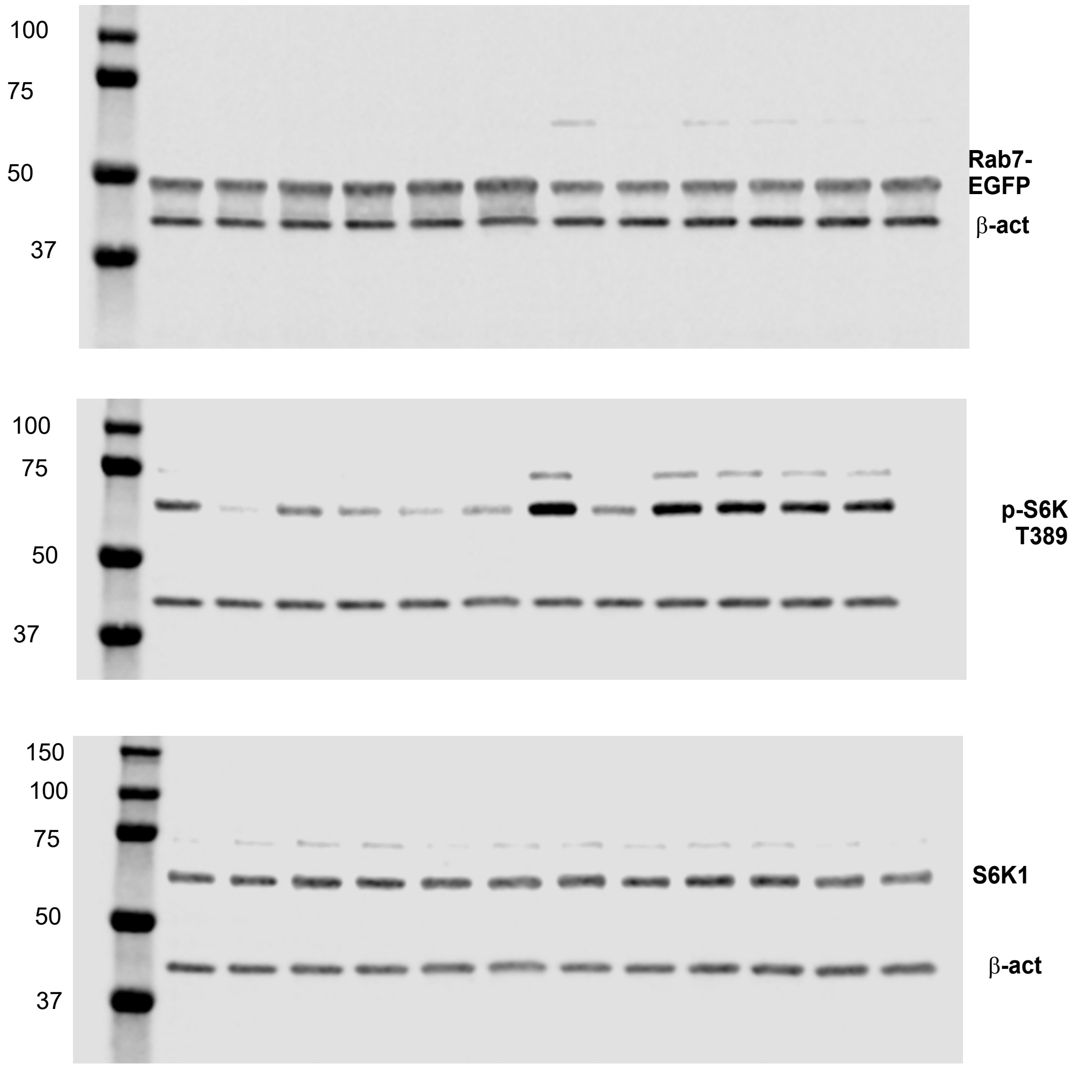
100
75
50
37
Rab7-
EGFP
β-act
100
75
50
37
p-S6K
T389
150
100
75
50
37
S6K1
β-act

Fig 3E

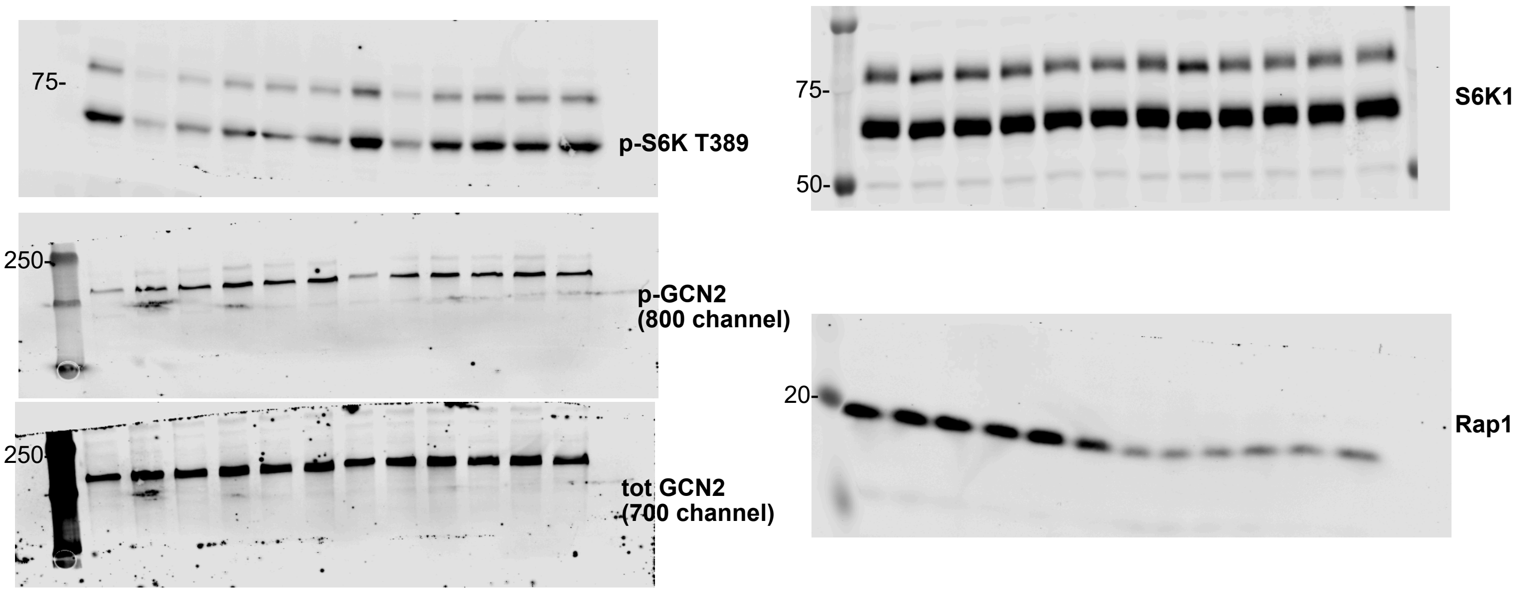

Fig 3F

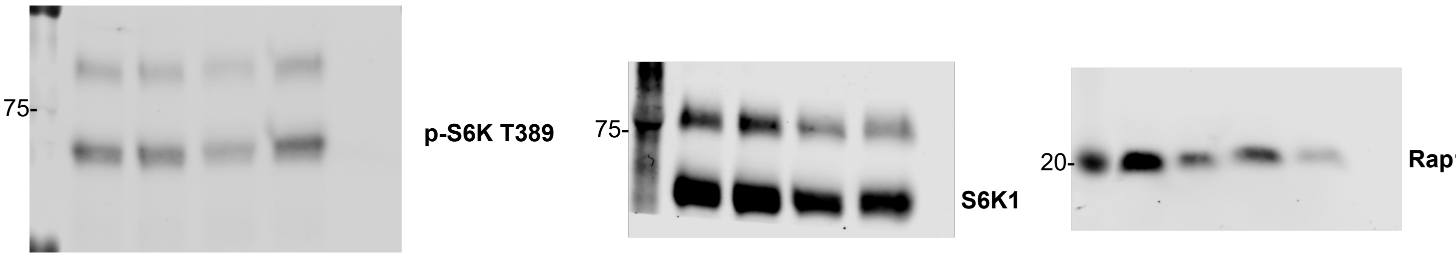

Fig 3I

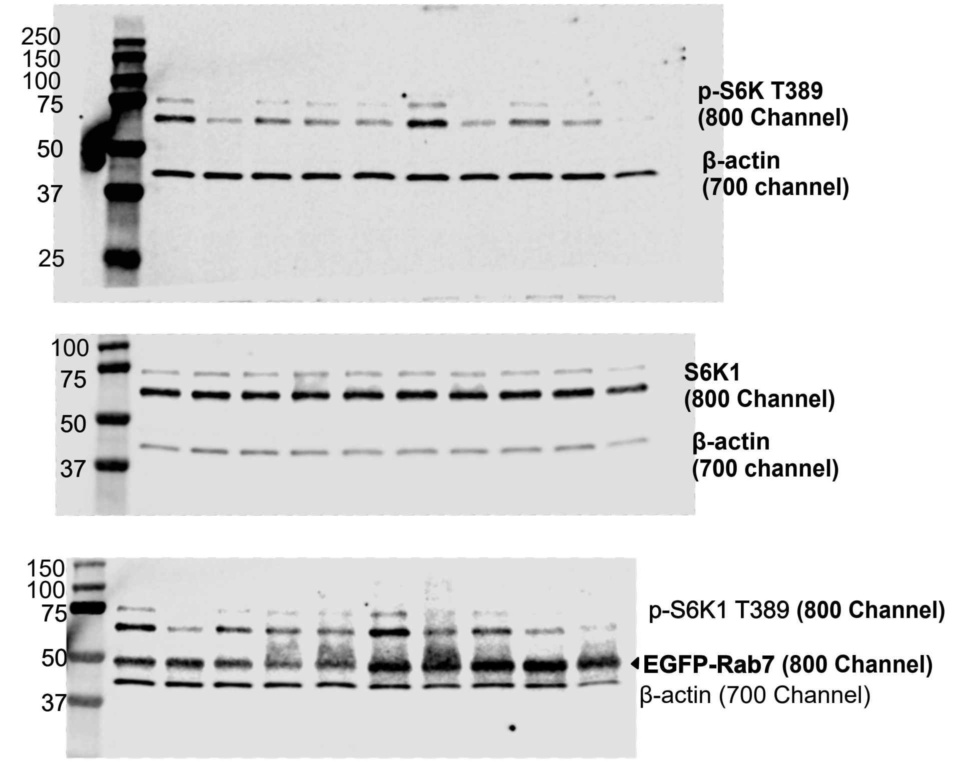

Fig 4A

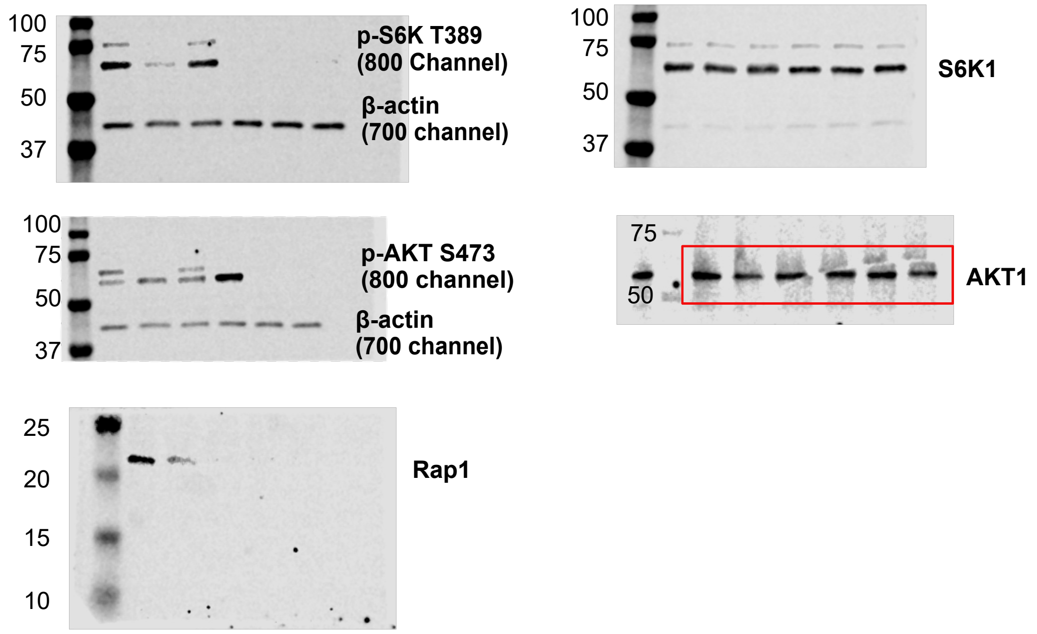

Fig 4B

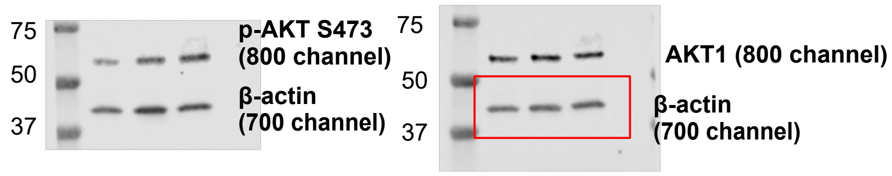

Fig 4C

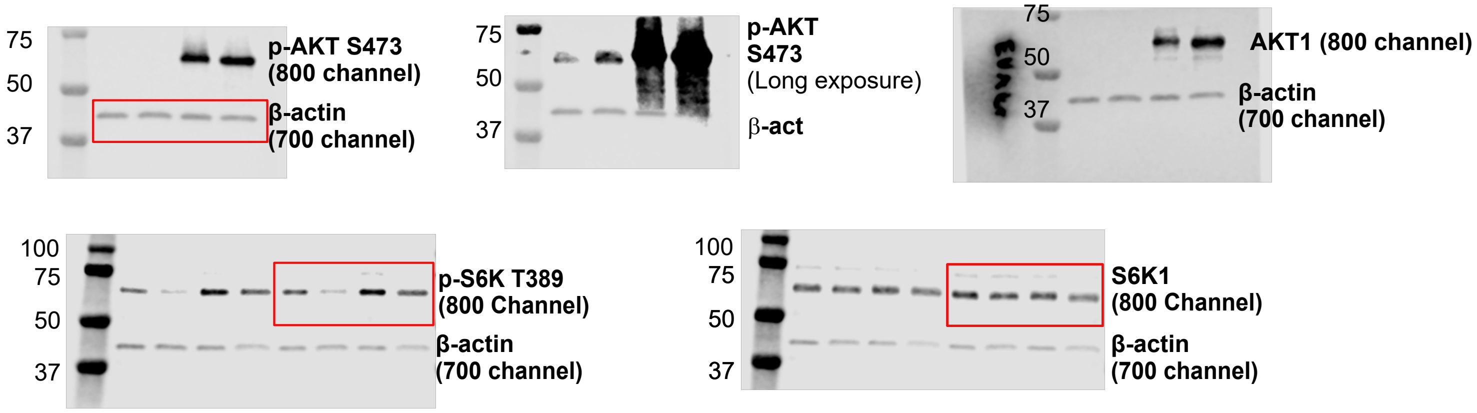

Fig 4D

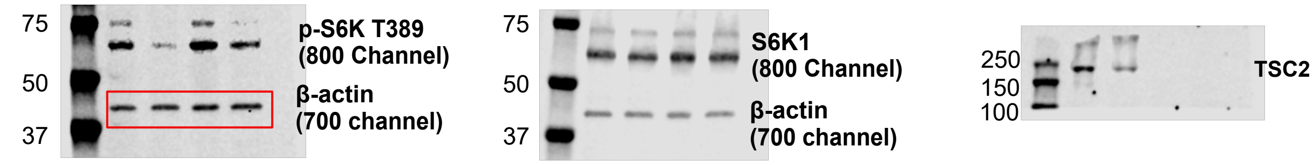

Fig 5A

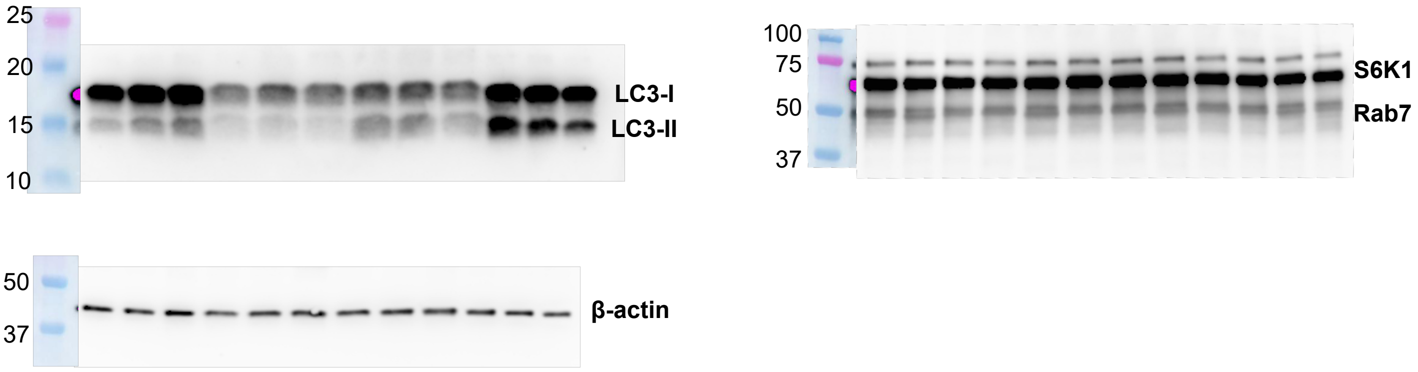

Fig 5F

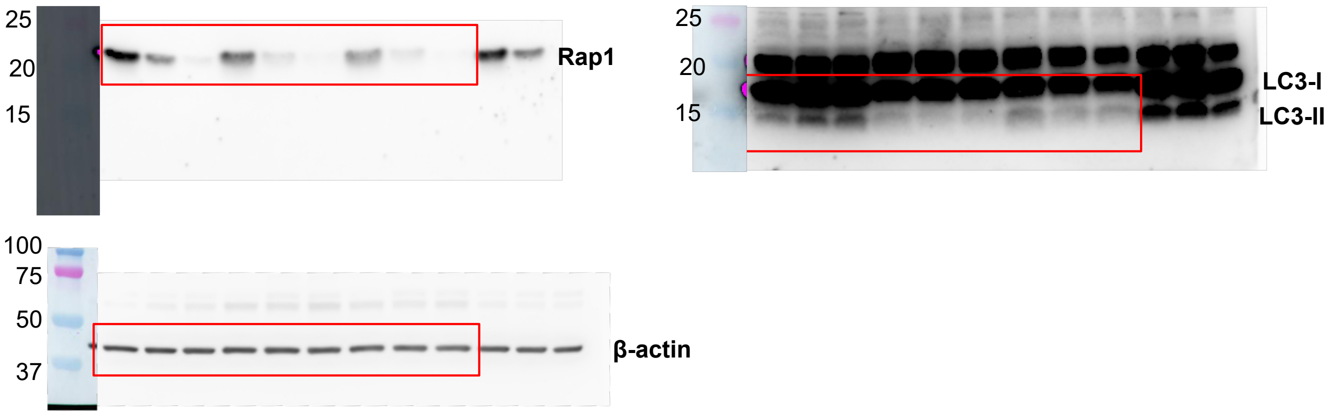

Fig 5G

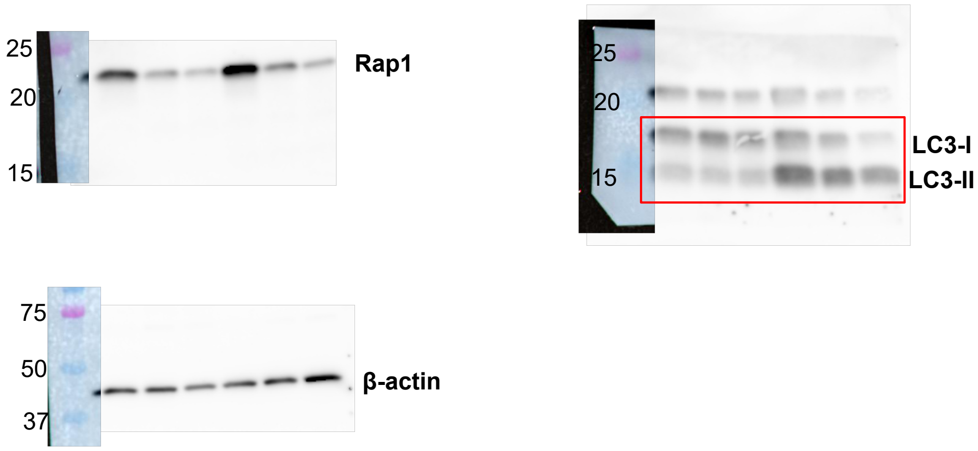

Fig 6A

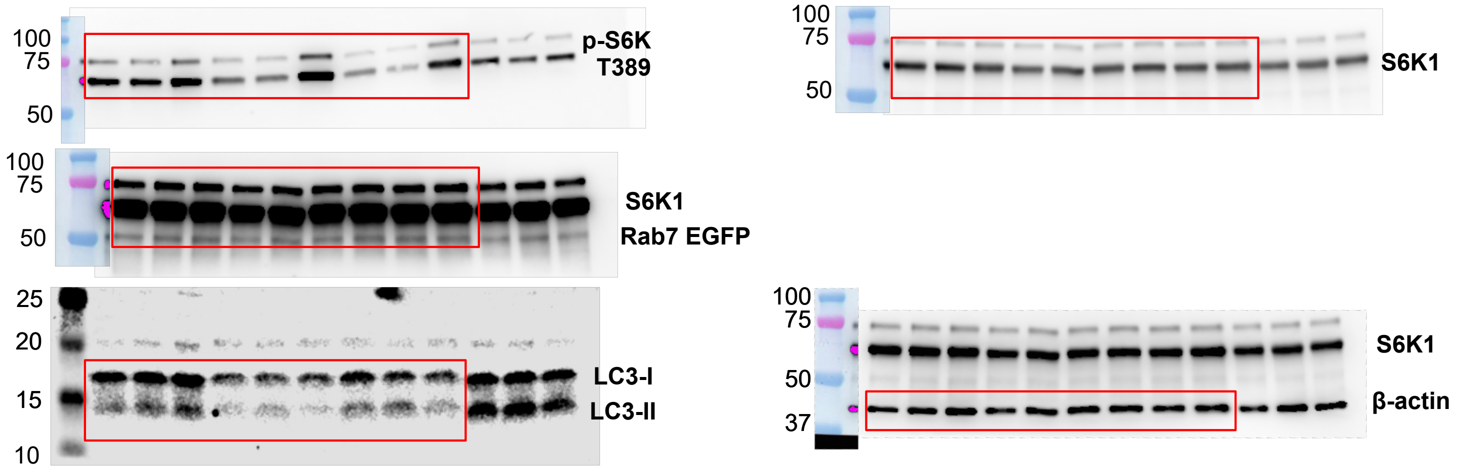

Fig 6B

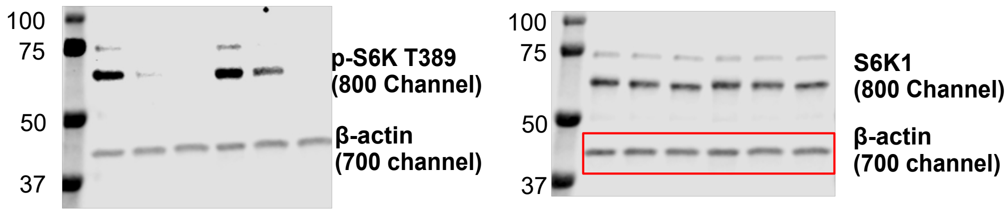

Fig 6C

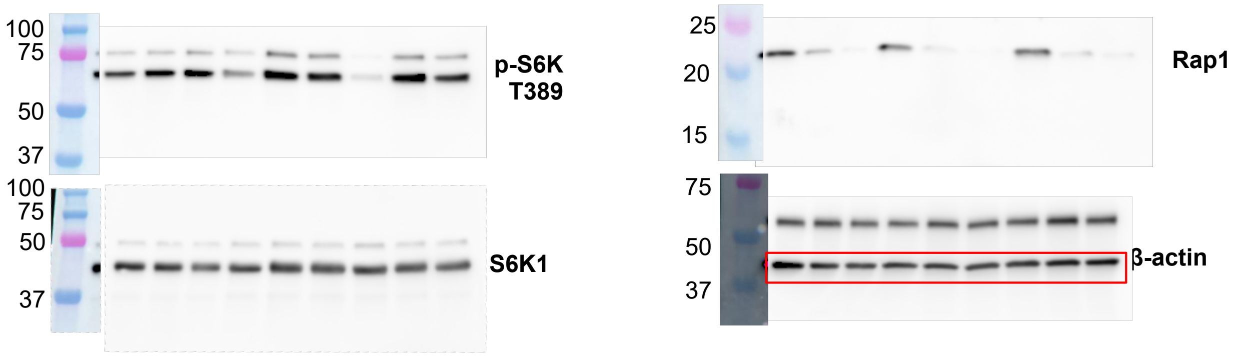

Fig 6D

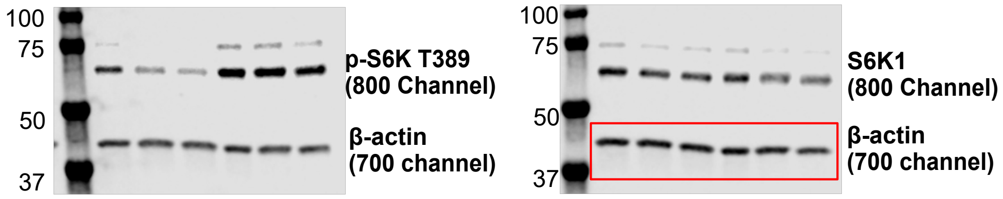

Fig 6E

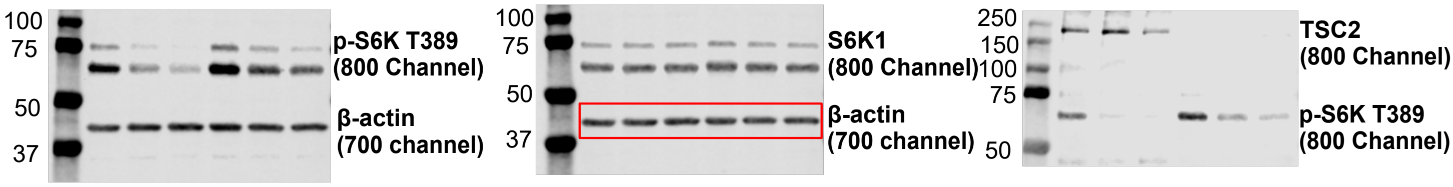

Fig 6F

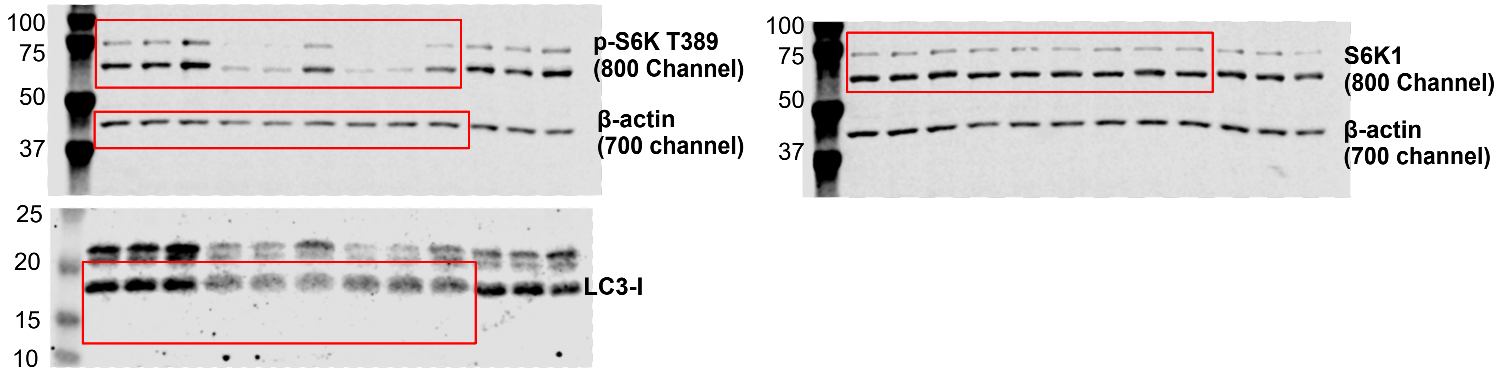

Fig S1C

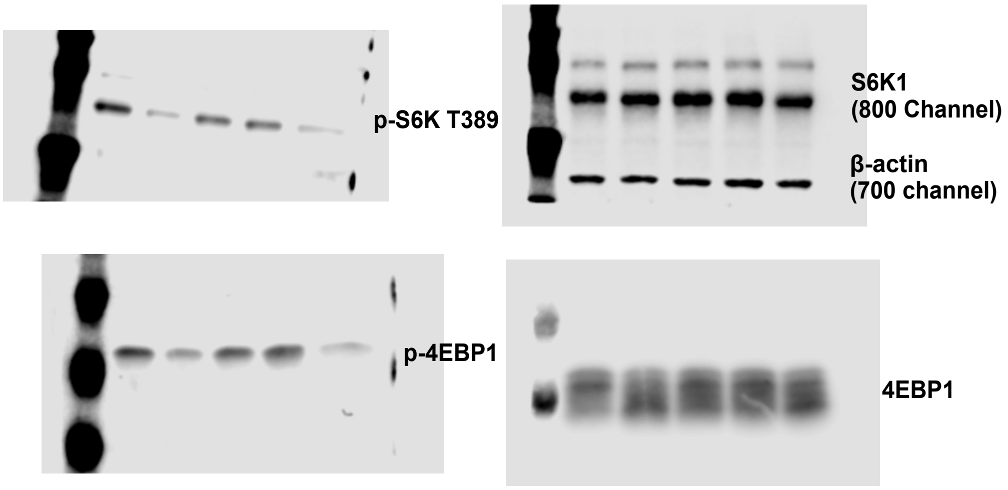

Fig S1D

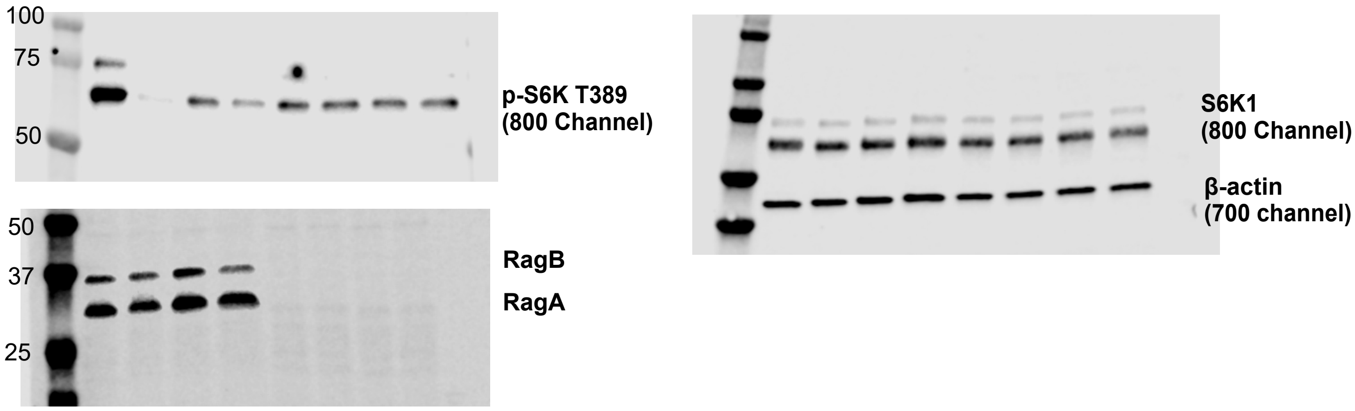

Fig S2C

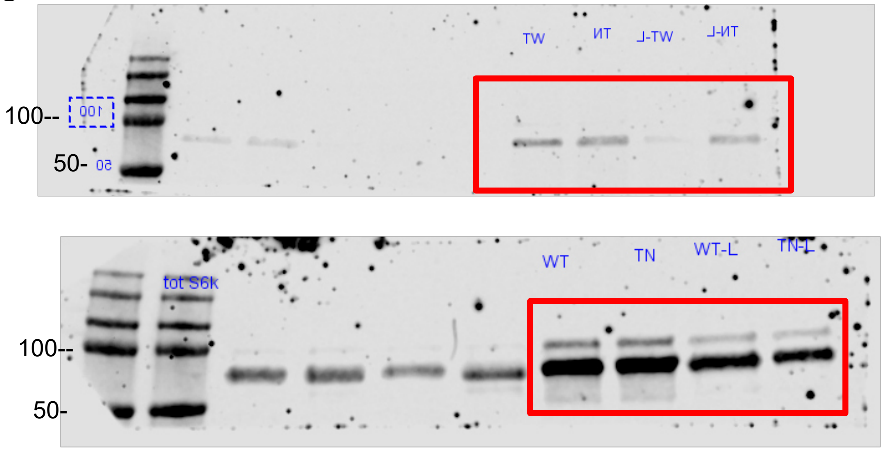

p-S6K T389

tot S6K1

Fig 3I

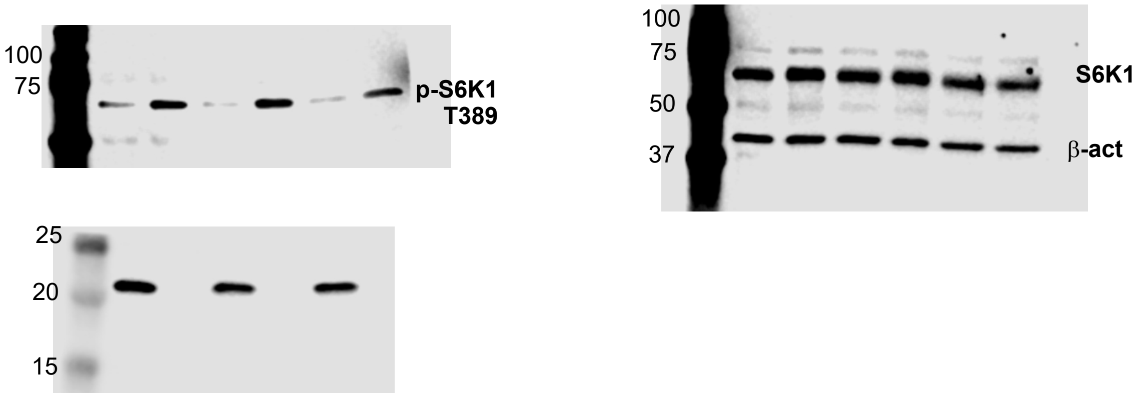
100
75
p-S6K1
T389
100
75
50
37
S6K1
β-act
25
20
15

Fig 3J

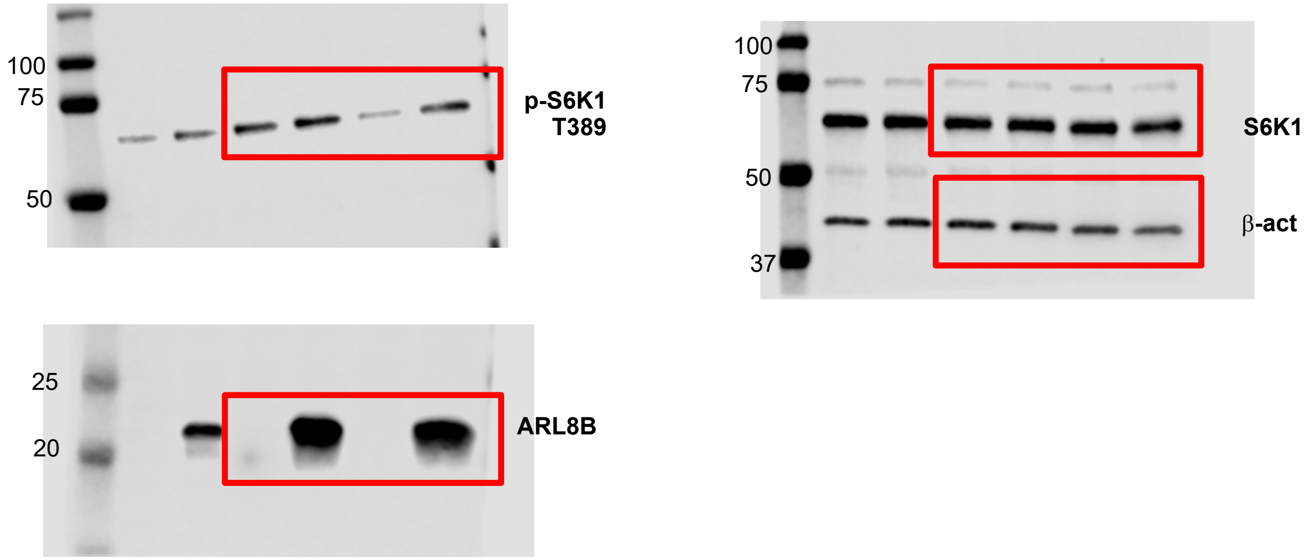
100
75
50
p-S6K1
T389
100
75
50
37
S6K1
β-act
25
20
ARL8B

Fig S4A

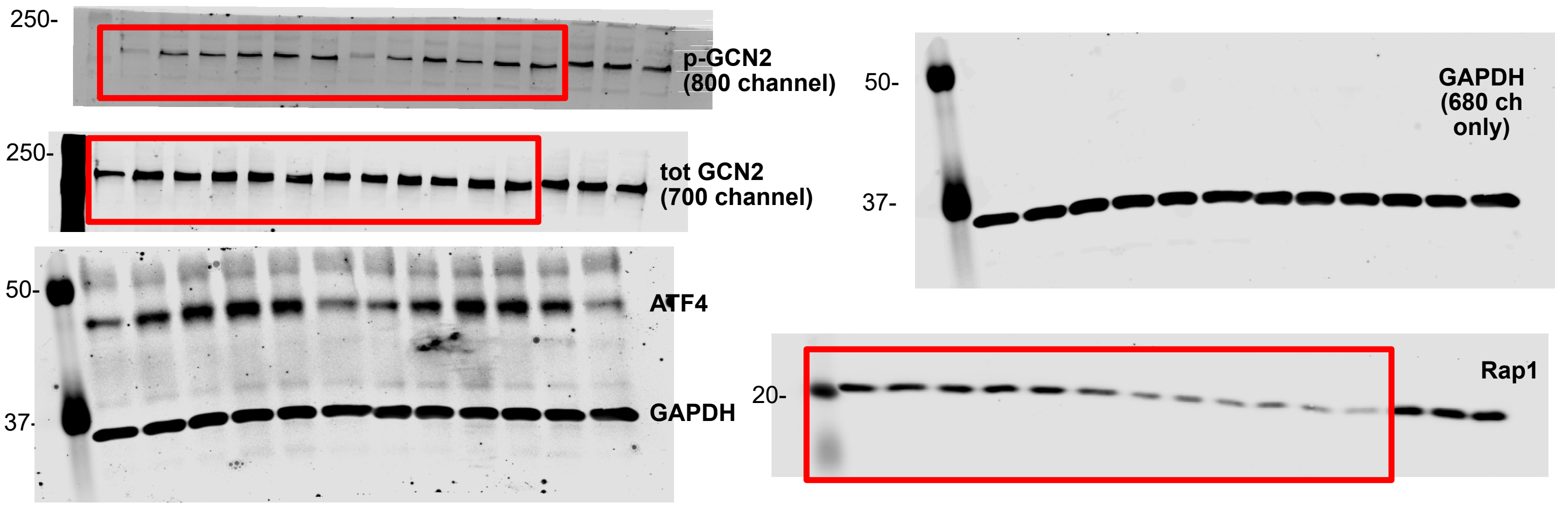

Fig S4B

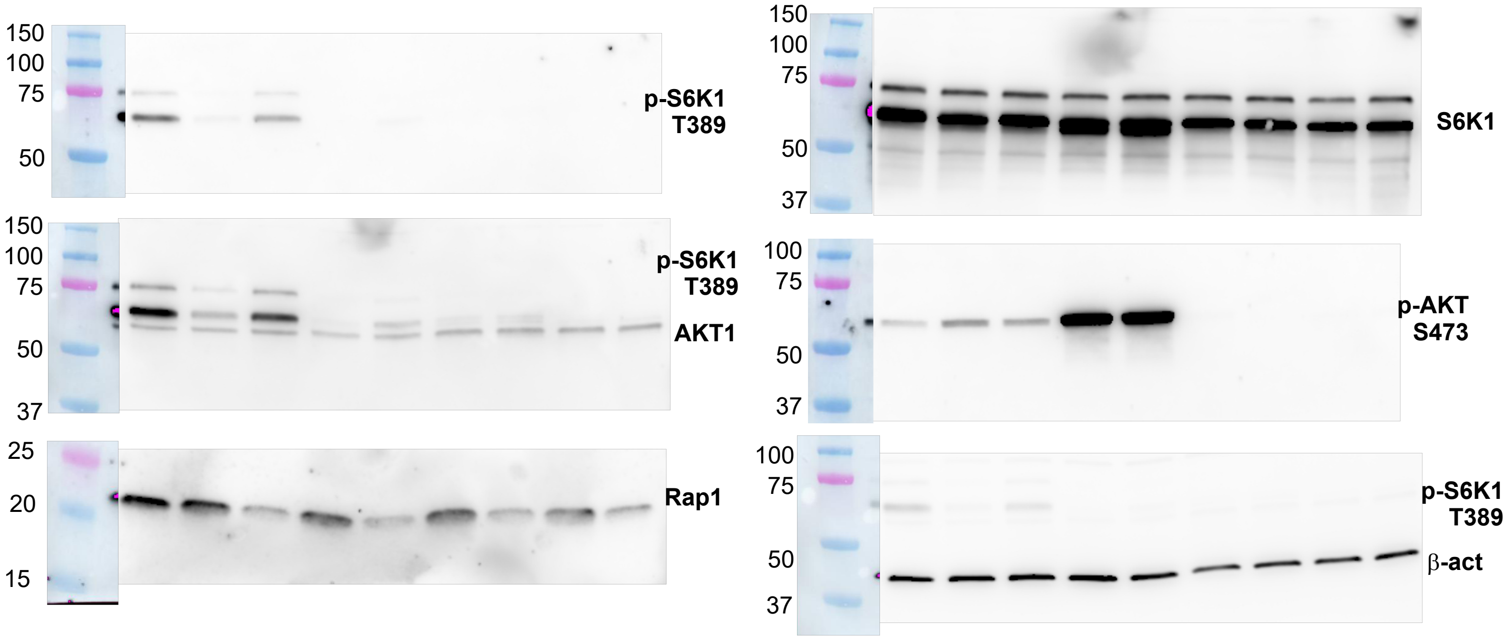

Fig S4E

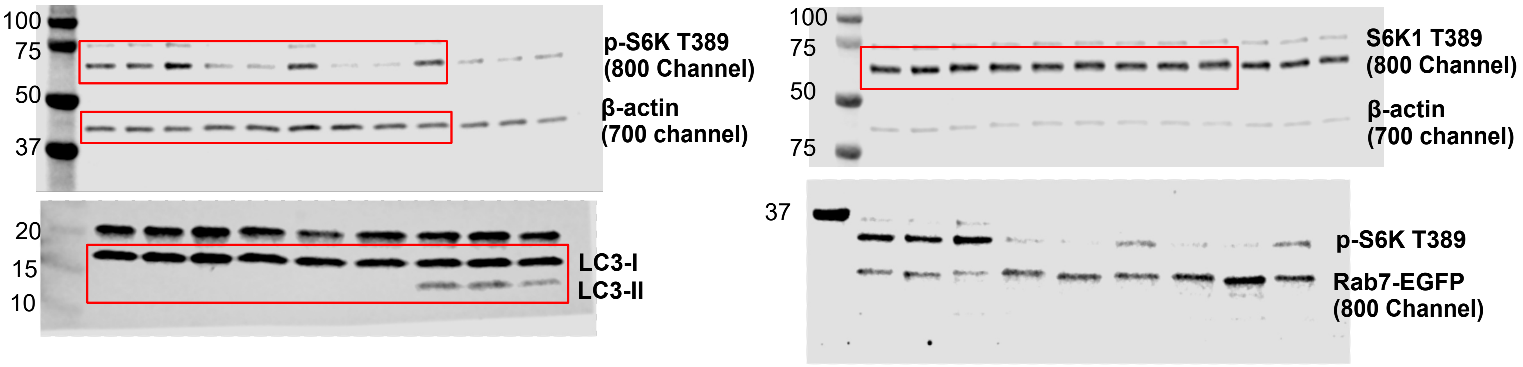

Fig S4F

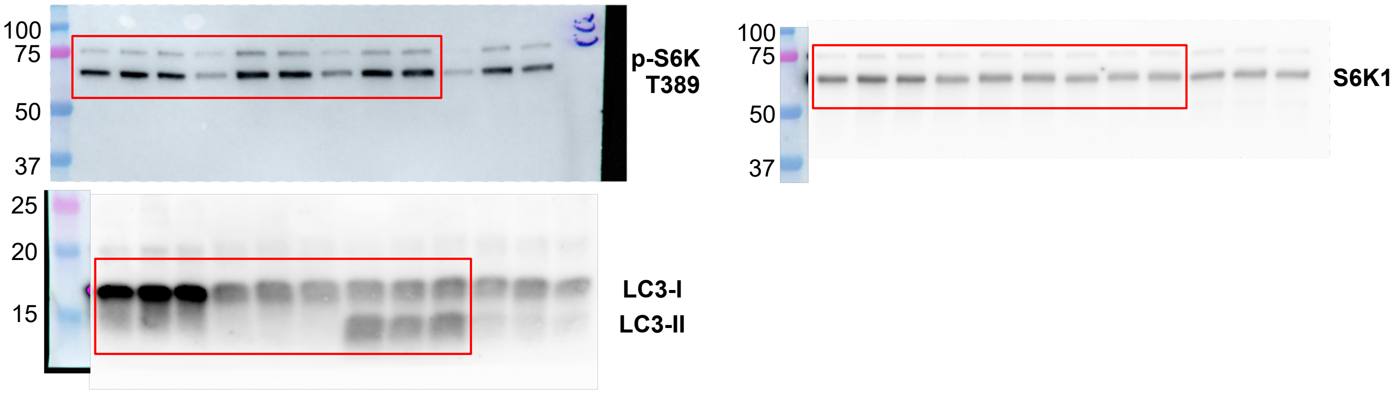

Supplement: Supplementary file 1 — Supplementary Material 1. [file 12964_2026_2659_MOESM1_ESM.pdf]
